# Hydrogen Sulfide Promotes Bone Homeostasis by Balancing Inflammatory Cytokine Signaling in CBS-Deficient Mice through an Epigenetic Mechanism

**DOI:** 10.1038/s41598-018-33149-9

**Published:** 2018-10-15

**Authors:** Jyotirmaya Behera, Kimberly E. Kelly, Michael J. Voor, Naira Metreveli, Suresh C. Tyagi, Neetu Tyagi

**Affiliations:** 1 Bone Biology Laboratory, Department of Physiology, School of Medicine, University of Louisville, Louisville, KY 40292 USA; 2 Department of Orthopaedic Surgery, School of Medicine, University of Louisville, Louisville, KY 40292 USA; 3 Department of Bioengineering, Speed School of Engineering, University of Louisville, Louisville, KY 40292 USA

## Abstract

Previously, we have shown hyperhomocysteinemia (HHcy) to have a detrimental effect on bone remodeling, which is associated with osteoporosis. During transsulfuration, Hcy is metabolized into hydrogen sulfide (H_2_S), a gasotransmitter molecule known to regulate bone formation. Therefore, in the present study, we examined whether H_2_S ameliorates HHcy induced epigenetic and molecular alterations leading to osteoporotic bone loss. To test this mechanism, we employed cystathionine-beta-synthase heterozygote knockout mice, fed with a methionine rich diet (CBS^+/−^ +Met), supplemented with H_2_S-donor NaHS for 8 weeks. Treatment with NaHS, normalizes plasma H_2_S, and completely prevents trabecular bone loss in CBS^+/−^ mice. Our data showed that HHcy caused inhibition of HDAC3 activity and subsequent inflammation by imbalancing redox homeostasis. The mechanistic study revealed that inflammatory cytokines (IL-6, TNF-α) are transcriptionally activated by an acetylated lysine residue in histone (H3K27ac) of chromatin by binding to its promoter and subsequently regulating gene expression. A blockade of HDAC3 inhibition in CBS^+/−^ mice by HDAC activator ITSA-1, led to the remodeling of histone landscapes in the genome and thereby attenuated histone acetylation-dependent inflammatory signaling. We also confirmed that RUNX2 was sulfhydrated by administration of NaHS. Collectively, restoration of H_2_S may provide a novel treatment for CBS-deficiency induced metabolic osteoporosis.

## Introduction

Osteoporosis, the most prevalent cause of bone fragility fractures, is characterized by low bone mass and structural deterioration of bone tissue. Osteoporotic fractures are associated with increased mortality and substantial economic expenses^1^. It affects 1 in 2 women and 1 in 5 men over age 50 and causes up to 9 million fractures per year worldwide^2–4^. Although the chances of developing osteoporosis are greater in women than in men due to post-menopausal oestrogen deficiency, the risk of osteoporotic mortality following fragility fracture is higher in men^5–7^. Hyperhomocysteinemia (HHcy) is reported to cause osteoporosis^1^. HHcy, a rare autosomal recessive disease, is characterized by marked increased plasma homocysteine (Hcy)^8^. HHcy is also recognized as a major risk factor for atherosclerotic vascular disease and cognitive impairment^9^. However, the pathophysiological consequence of HHcy in the skeletal system, especially its osteoporotic phenotype, is still unknown. A human-based cohort study reported that elevated plasma Hcy was detrimental to developing osteoporotic fractures^1^. Also, a few *in vivo* and *in vitro* studies have revealed that Hcy is associated with collagen crosslinking in bone, leading to bone architectural deterioration^10–12^. The recent *in vitro* study suggests that bone resorption and turnover rate were higher in HHcy through an elevated oxidative imbalance^13^. Therefore, a better understanding of the complex molecular regulatory pathways controlling the process of bone deterioration, is crucial to improve our understanding of skeletal development.

Histone deacetylases (HDACs) are important enzyme complexes that cause various physiological processes. They are known to act as a transcriptional corepressors that epigenetically control gene transcription by removing acetyl groups from lysine side chains of nucleosomal histone tails, leading to chromatin condensation and gene repression^14–16^. The mammalian HDACs are classified into 4 classes on the basis of their structure and functions^16^. Class 1 HDACs (HDAC1, HDAC2, HDAC3 and HDAC8) have high enzymatic activity and are widely localized to the nucleus. However, HDAC3 has also been located at plasma membranes^15^. Class II HDACs (HDAC4, HDAC5, HDAC6, HDAC7, HDAC9 and HDAC10) have low intrinsic enzymatic activity and have been found to be localized between the nucleus and cytoplasm, as well as modulate temporal and spatial gene expression patterns. Class III HDACs [sirtuins (SIRTs)] require nicotinamide adenine dinucleotide (NAD+) for their catalytic activity. Lastly, class IV HDACs (HDAC11), is a special class which shares properties of both class I and class II HDACs^17^. HDACs can deacetylase a number of proteins posttranscriptionally, including runt-related transcription factor 2 (RUNX2), nuclear factor kappa-light-chain-enhancer of activated B cells (NF-κB) and tumor protein p53 (P53)^18–20^. Small molecules that target HDACs have been used to treat a number of conditions such as neurocognitive impairment and arthritis^21,22^. Also, long-term inhibition of HDACs activity in humans increases risk of fracture and reduces bone mineral density in mice^23–25^. Recent work^16^ has suggested that HDAC3 inhibition activates inflammatory cytokine signaling by degradation of cartilage, thereby increasing histone acetylation in chromatin. Therefore, it is essential to understand the pathological role of HHcy on HDAC activity in bone formation.

Hydrogen sulfide (H_2_S) is a novel gasotransmitter endogenously produced by mammalian tissues and mediates diverse physiological functions^26,27^. Abnormal H_2_S production is associated with several pathophysiological outcomes such as Alzheimer’s disease, hypertension and diabetes^28–30^. H_2_S is physiologically produced L-cysteine, catalyzed by two pyridoxal-5′-phosphate-dependent enzymes, termed cystathionine β-synthase (CBS) and cystathionine γ-lyase (CSE)^31^. CBS is a predominant H_2_S-generating enzyme in the brain, nervous system and bone, whereas CSE is mainly expressed in the vascular system and pancreas^28,31–33^. H_2_S was found to be a protective molecule against oxidative stress by restoring redox homeostasis and inducing antioxidant transcription factor Nrf2^34,35^. Additionally, H_2_S acts as an anti-inflammatory agent and protects against leukocyte-mediated inflammation^36^. A recent epidemiological study reported that patients suffering from HHcy have an increased risk of osteoporotic fracture^1,37,38^. In addition to the elevated level of Hcy, CBS deficient patients also display decreased levels of plasma H_2_S^31^.

The report suggests that H_2_S is required for maintenance of bone mass by controlling bone marrow mesenchymal stem cells (BMMSCs) function via Ca^2+^ channel sulfhydration^31^. Additionally, H_2_S deficiency causes the osteopenia phenotype in mice by dysregulating BMMSCs function. CBS-deficient mice typically produce the HHcy phenotype, which ultimately promotes resident monocyte inflammation and blood brain barrier damage^39,40^. This prompted us to gain an understanding as to whether CBS-deficiency induces BMMSCs inflammation and to discover the epigenetic role of H_2_S in HHcy induced bone loss in CBS-deficient mice. In this study, we report that H_2_S in BMMSCs maintains bone homeostasis via a HDAC3 dependent manner. Furthermore, our study provides evidence that HDAC3 normally attenuates inflammatory cytokine expression during bone formation. We also show that pharmacological restoration of H_2_S could prevent HHcy induced histone acetylation that leads to bone loss in CBS-deficient mice.

## Results

### Phenotype of CBS^+/−^ Mice Derived BMMSCs and Effect on H_2_S production *in vivo* and *in vitro*

**Figure 1:**
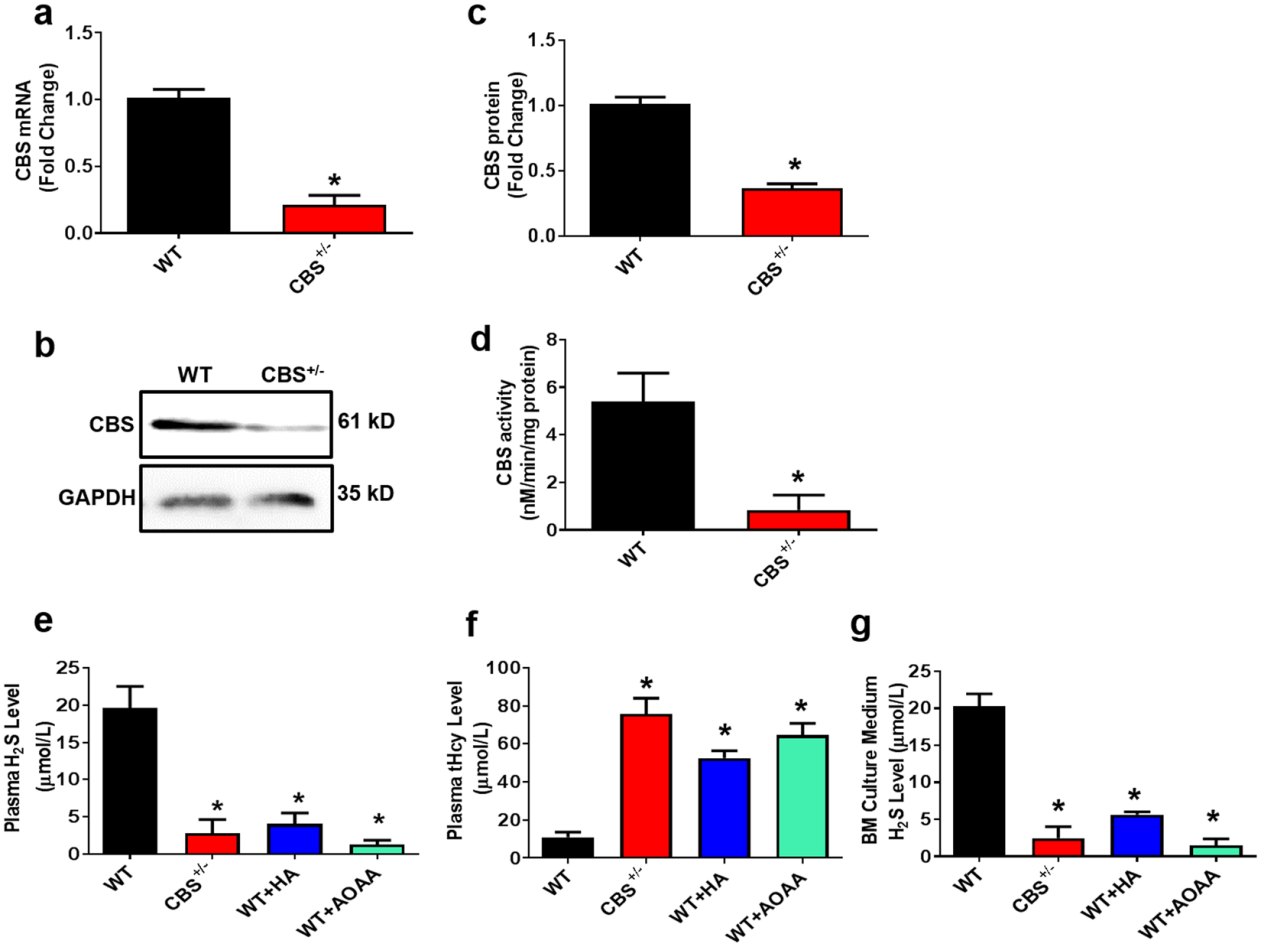
CBS is essential for physiological levels of H_2_S production. (**a**) Mouse BMMSCs expressed CBS, as assessed by RT-PCR. (**b**,**c**) Western blot analysis confirmed that BMMSCs expressed CBS. (**d**) CBS activity in experimental mice. (**e**) Plasma H_2_S levels were measured in experimental mice. The H_2_S level was decreased in the CBS^+/−^ group in comparison with WT. However, its level was further reduced by i.p. injection of CBS inhibitor hydroxylamine (HA) or aminooxyacetic acid (AOAA). (**f**) Biochemical assay confirmed the increased tHcy level in the plasma of CBS^+/−^, HA or AOAA mice group. (**g**) Mouse BMMSCs culture supernatant contained approximately 20.3 ± 1.675 μM of H_2_S. H_2_S levels in BMMSCs culture supernatants were downregulated in the CBS^+/−^ mice and also CBS inhibitor HA or AOAA treatment groups for 8 weeks to WT mice respectively (2.4 ± 1.533 μM, 5.6 ± 0.367 μM, and 1.5 ± 0.867 μM). Experiments were repeated three times. Data are expressed as mean ± SEM. n = 5 mice per group. *p < 0.05 compared with the WT mice.

H_2_S has an important physiological role in a variety of cells and is enzymatically produced by CBS and CSE. It can be found as free H_2_S or in the form of sulfur^31,41^. In investigating whether CBS ablation in mice affects the systemic level of H_2_S and its production in bone, 16-week-old female CBS^+/−^ were used. Interestingly, we found that CBS expression was lower in BMMSCs, as assessed by RT-PCR and Western blotting (Fig. 1a–c). Compared to wild-type (WT) mice, CBS^+/−^ mice displayed a decreased level of CBS activity (Fig. 1d). Treatment of the CBS inhibitor hydroxylamine (HA) or aminooxyacetic acid (AOAA) to WT mice derived plasma significantly decreased H_2_S levels (Fig. 1e). To further study the HHcy condition in CBS^+/−^ mice, we measured the plasma level of total Hcy (tHcy). The results showed that the plasma level of tHcy was significantly higher in CBS^+/−^ mice in comparison to WT and CBS inhibitor (both HA and AOAA) treated mice (Fig. 1f). Also, BMMSCs were able to produce H_2_S in culture supernatant at a level of 20.3 ± 1.675 μM. However, this level was actively lowered in CBS^+/−^ mice and other experimental groups respectively (2.4 ± 1.533 μM, 5.6 ± 0.367 μM, 1.5 ± 0.867 μM) (Fig. 1g). The details of BMMSCs characterization and genotype analysis of CBS^+/−^ mice were checked for targeted disruption of the CBS^+/−^gene, as shown in Supplementary Fig. 1. These findings suggest that CBS is essential for the production of H_2_S in both *in vitro* and *in vivo* conditions (Fig. 1).

### H_2_S Supplementation Ameliorated HHcy-Induced Redox Homeostasis

**Figure 2:**
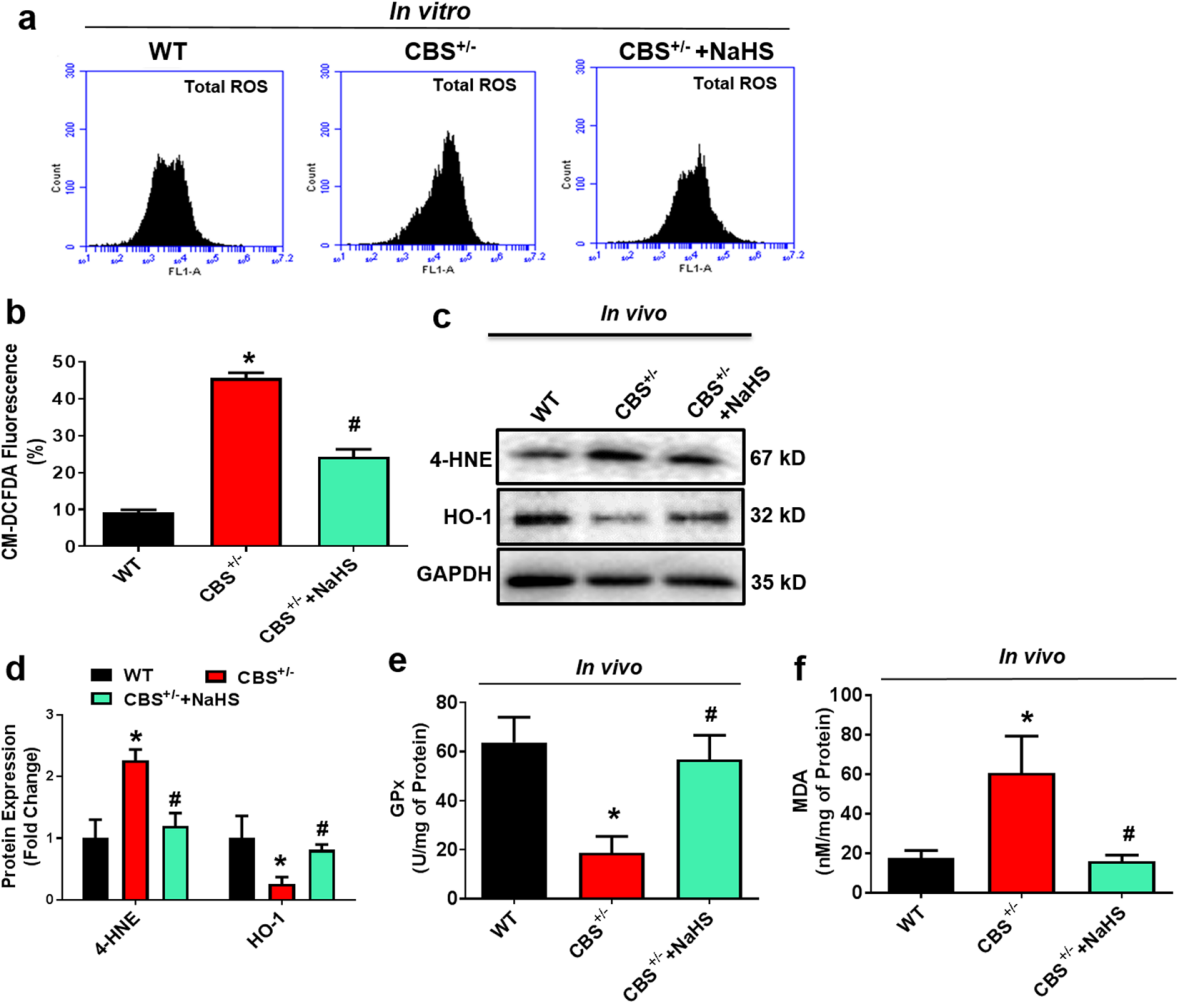
Administration of H_2_S rescued the CBS-deficiency induced oxidative stress. (**a**,**b**) Flow cytometry analysis confirmed that ROS production was increased in CBS-deficient BMMSCs culture, as analyzed by DCFDA flow cytometry. (**c**,**d**) Western blot analysis of NOX-4 and HO-1 expression in the whole femur bone extracts. (**e**) Cellular antioxidant enzyme GPx activity in whole femur bone extracts. (**f**) Lipid peroxidation in the bone marrow (BM) plasma was assessed by MDA assay. Experiments were repeated three times. Data are expressed as mean ± SEM. n = 5 mice per group. *p < 0.05 compared with the WT mice, ^#^p < 0.05 compared with the CBS^+/−^ mice.

To examine the protective effect of H_2_S on the HHcy induced oxidative damage in the bone milieu, several vital indices of oxidative stress were determined. We measured total reactive oxygen species (ROS) production in BMMSCs cells (BM cells isolated from experimental mice) by flow cytometry after staining with oxidative fluorescent dye 2′,7′-dichlorofluorescin diacetate (DCF-DA). The level of total ROS was indeed increased in BMMSCs of CBS^+/−^ mice, as assessed by flow cytometry analysis and this increase in total ROS was attenuated by sodium hydrosulfide (NaHS); a H_2_S donor (Fig. 2a,b). Since the balance between oxidant and anti-oxidant is important for bone homeostasis, the levels of heme oxygenase-1 (HO-1), glutathione peroxidase (GPx), 4-Hydroxynonenal (4-HNE), and malondialdehyde (MDA) were measured to assess oxidative damage in the bone and determine whether H_2_S protects against this type of damage in the bone milieu in the experimental group. As shown in Fig. 2c,d the protein expression of 4-HNE was higher in femur bone extract of CBS^+/−^ mice compared to WT. However, HO-1, expression was indeed decreased in CBS^+/−^ mice (Fig. 2c,d). In addition, we also observed antioxidant enzyme, GPx, activity in whole femur extracts by ELISA in the experimental condition. The results showed that GPx enzyme activity was decreased in CBS^+/−^ mice in comparison with WT (Fig. 2e). However, supplementation of NaHS normalized the above changes in the CBS^+/−^ mice. As an index of oxidative damage, the level of lipid peroxidation product, MDA, was measured and was found to be significantly higher in CBS^+/−^ mice than in WT mice (Fig. 2f). These findings suggest that administration of NaHS could maintain the redox homeostasis in bone milieu *in vivo* (Fig. 2).

### Effect of H_2_S on BMMSCs Transcriptome and Chromatin Landscape via HDAC3 Dependent Mechanism *in vivo*

**Figure 3:**
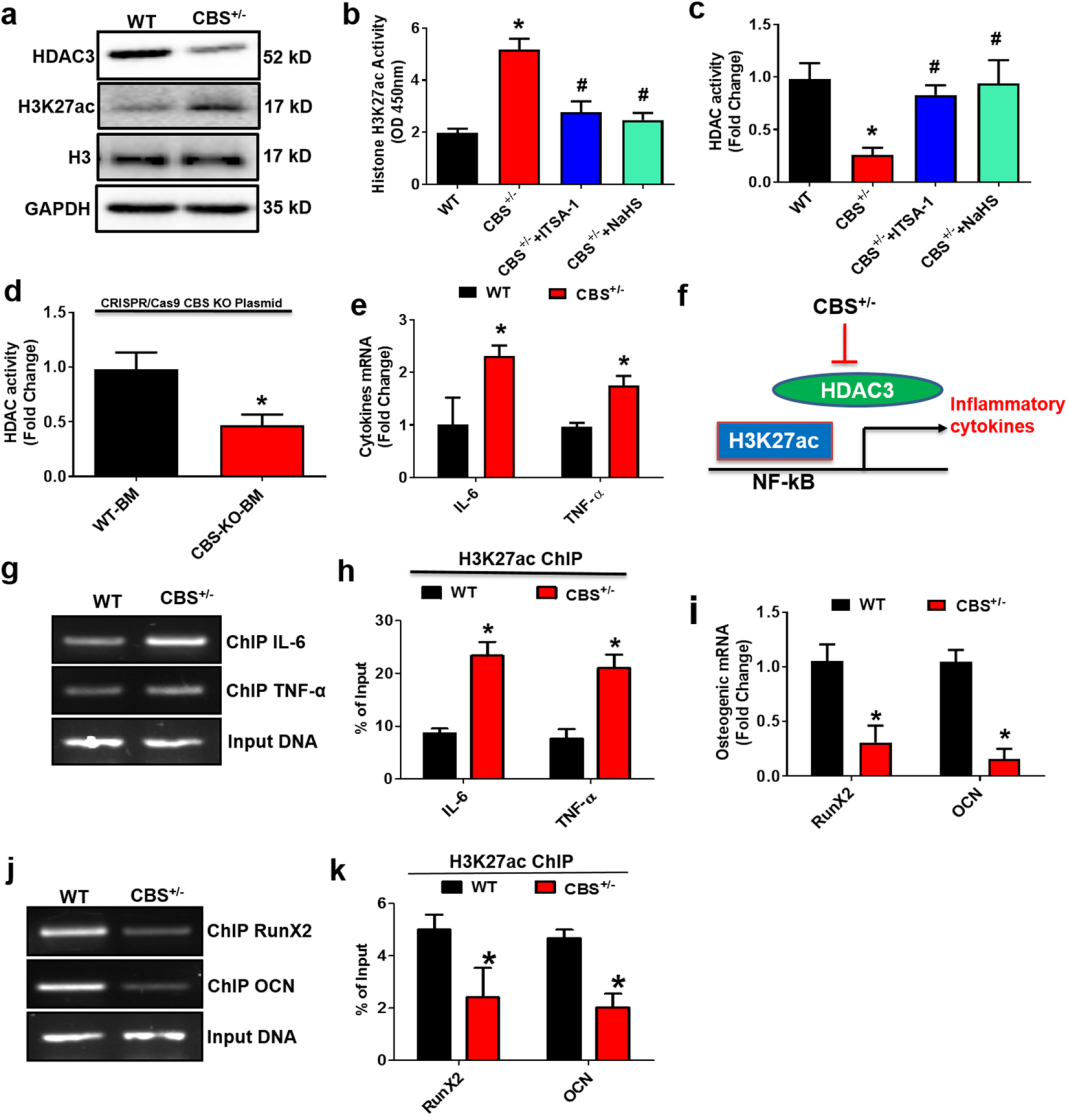
H_2_S-Deficient mice negatively regulate global gene expression and histone acetylation. (**a**) Western blot analysis of the indicated proteins (HDAC3, H3K27ac and H3) derived from femur bone extract of CBS^+/−^ mice. (**b**) Global Acetyl Histone H3K27 Quantification Kit confirmed that overall histone acetylation (H3K27) was indeed increased in femur bone extracts of CBS^+/−^ mice. (**c**) Histone Deacetylase (HDAC) Activity was decreased in CBS^+/−^ mice as assessed by HDAC activity fluorometric assay kit. (**d**) Histone deacetylase (HDAC) activity of BMMSCs derived from WT mice, were transfected with CBS CRISPR/Cas9 Knockout (KO) plasmid. (**e**) Differential gene expression of representative inflammatory genes in femur bone extracts of CBS^+/−^ mice. (**f**) Diagrammatic representation of H3K27ac binding to target genes of inflammatory cytokines. (**g**,**h**) Chromatin immune precipitation (ChIP) assay was performed assessing H3K27ac binding into the promoter of IL-6 and TNF-α. The results are expressed as the percentage of input. (i) Differential gene expression of representative osteogenic genes in femur bone extracts of CBS^+/−^ mice. (**j**,**k**) ChIP assay was performed assessing H3K27ac binding into the promoter of OCN and RunX2. The results are expressed as the percentage of input. Experiments were repeated three times. Data are expressed as mean ± SEM. n = 5 mice per group. *p < 0.05 compared with the WT mice, ^#^p < 0.05 compared with the CBS^+/−^ mice.

Histone deacetylase 3 (HDAC3) is a nuclear enzyme, which plays a key role in skeletal development^16^. To understand the role of HDAC3 in bone formation under the HHcy condition in CBS^+/−^ mice. We evaluated the levels of the HDAC3, lysine acetylation of histone H3 chain (H3K27ac) and histone H3 in the whole femur extracts of the experimental groups. The protein western blot study revealed that HDAC3 expression was lower in the CBS^+/−^ mice as compared to WT (Fig. 3a). However, in turn, the lysine acetylation of histone H3 (H3K27ac) level was indeed increased in CBS^+/−^ mice (Fig. 3a). ELISA confirmed these results by measuring the activity of HDAC and H3K27ac in genomic DNA isolated from mouse femur bone (Fig. 3b,c). However, administration of Inhibitor-1 of Trichostatin A (ITSA-1) or NaHS in the CBS^+/−^ mice was able to reverse the above changes in the experimental group (Fig. 3b,c). To re-confirm the role of HHcy in the imbalance of HDAC3 and H3K27ac activity, BMMSCs derived from WT mice, were transfected with CBS CRISPR/Cas9 Knockout (KO) plasmid. The results showed that decreased HDAC activity was observed in CBS knockout BMMSCs (Fig. 3d). These data provide evidence that CBS deficiency induces HHcy, which causes alternation of chromatin landscapes by attenuating HDAC activity and is shown to be prone to mediate inflammation.

Because it is unknown whether HHcy affects HDAC activity leading to alternations of transcriptional gene expression in BMMSCs, quantitative gene expression profiling confirmed that mRNA levels of inflammatory cytokines (Interleukin 6 (IL-6) and tumor necrosis factor alpha (TNF-α)) were up-regulated in CBS^+/−^ mice (Fig. 3e). In addition, osteogenic gene expressions (RunX2 and osteocalcin (OCN)) were down-regulated in CBS^+/−^ mice derived BMMSCs culture (Fig. 3i). This provides the understanding that CBS deficiency induced HHcy, causing inflammation by attenuating HDAC activity.

We performed a ChIP-qPCR analysis to investigate the abundance and binding affinity of H3K27ac in the promoter regions of selective pro-inflammatory genes (IL-6 and TNF-α) in HHcy conditions (Fig. 3f). Interestingly, HHcy increased the enrichment of H3K27ac at the promoters of IL-6 and TNF-α as compared with WT (Fig. 3g,h). This study also examined the HHcy mediated occupancy of H3K27ac at the promoter regions of OCN and RunX2 in the experimental conditions. The data suggest that HHcy caused reduced occupancy of H3K27ac at the promoters of OCN and RunX2 as compared with WT (Fig. 3j,k). However, the level of H3K27ac at the input DNA was not significantly different with or without HHcy (Fig. 3). Together, our work demonstrates that HDAC3 inhibition suppresses osteogenic gene expression and further upregulates the histone acetylation dependent inflammatory cytokine expression.

### Effect of H_2_S on HHcy Induced NF-κB Acetylation Dependent Pro-Inflammatory Signaling *in vivo*

**Figure 4:**
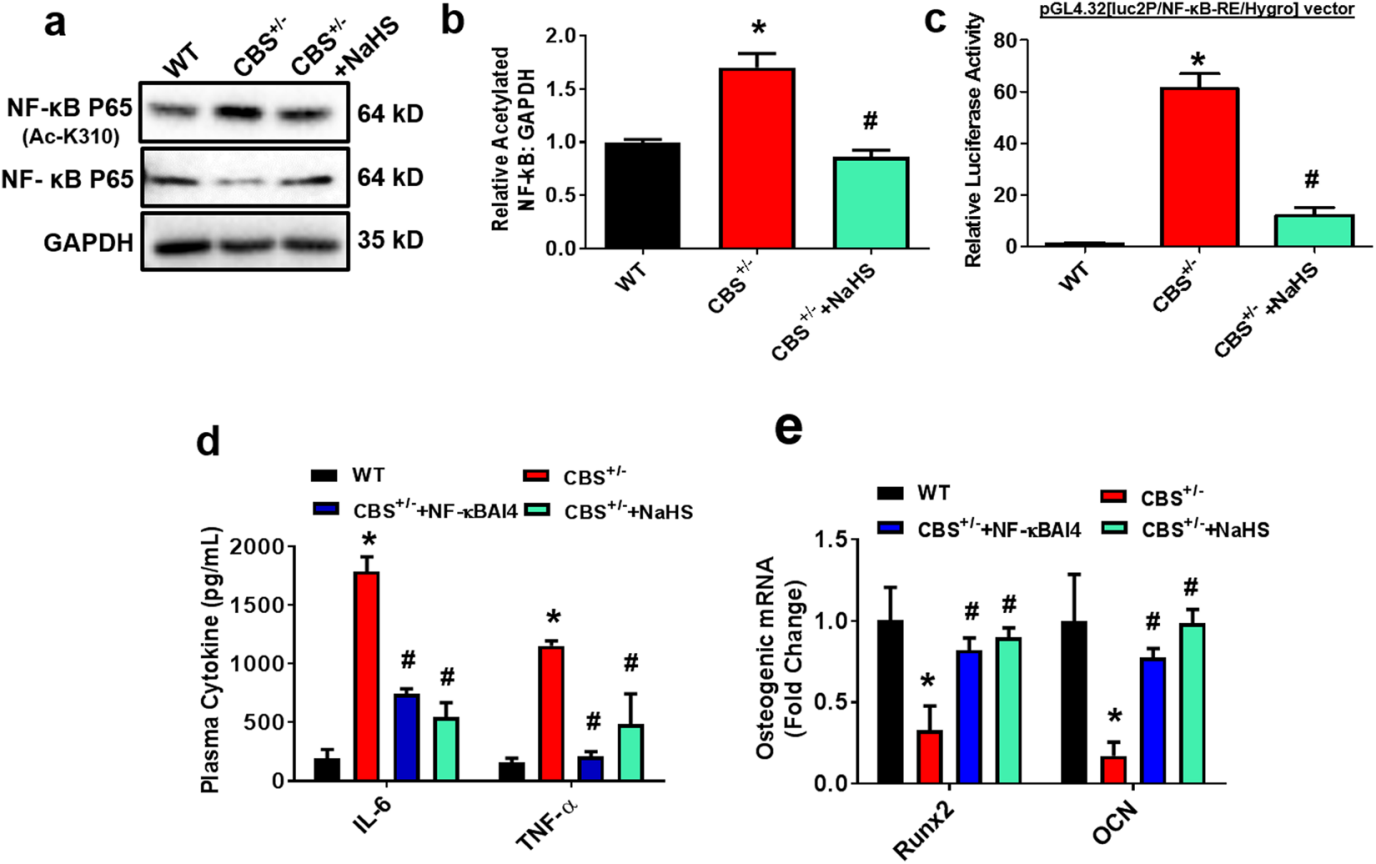
Administration of NaHS ameliorates HHcy induced NF-κB acetylation dependent proinflammatory response in CBS^+/−^ mice. (**a**,**b**) Protein western blot analysis confirmed that NF-κB acetylation (p65) activity was present at the K310 region of CBS-deficient mice. (**c**) CBS^+/−^ mice derived BMMSCs culture was transfected with pGL4.32[luc2P/NF-jB-RE/Hygro] vector, for the first 48 hours. Increased luciferase activity was observed in CBS^+/−^ mice derived BMMSCs. (**d**) Plasma inflammatory cytokine levels were measured in the experimental mice using ELISA. (**e**) qPCR was performed for the osteogenic gene transcripts: OCN and RunX2. Experiments were repeated three times. Data are expressed as mean ± SEM. n = 5 mice per group. *p < 0.05 compared with the WT mice, ^#^p < 0.05 compared with the CBS^+/−^ mice.

Next, we evaluated NF-κB acetylation and its role in inflammatory signaling. Our data demonstrated that there was increased NF-κB p65 acetylation at the K310 region in the femur bone extracts of CBS^+/−^ mice as compared to WT (Fig. 4a,b). Interestingly, CBS^+/−^ mice that received NaHS treatment showed a significant reduction in NF-κB p65 acetylation (Fig. 4a,b). We further examined the nuclear NF-κB transcription factor activity in CBS^+/−^ derived BMMSCs by a dual-luciferase reporter assay system. Experimental mice derived BMMSCs were transfected with the pGL4.32[luc2P/NF-κB-RE/Hygro] vector encoding the firefly luciferase reporter gene (luc2P) driven by five copies of an NF-κB enhancer element for 48 hours. As shown in the figure (Fig. 4c), there was significantly increased NF-κB transcription factor activity in BMMSCs of CBS^+/−^ mice as compared to WT (Fig. 4c). However, this effect was reversed with treatment of NaHS in CBS^+/−^ mice. We demonstrated whether NF-κB affects the inflammatory response as well as modulation of osteogenic gene expression in the experimental groups. Using ELISA assay, we determined the levels of IL-6 and TNF-α expression in plasma of experimental mice. The data suggested that inflammatory cytokines such as IL-6 and TNF-α were indeed increased in plasma of CBS^+/−^ mice as compared to WT. In contrast, treatment with NF-κB Activation Inhibitor IV (NF-κBAI4) and NaHS in the CBS^+/−^ mice prevented the promotion of inflammatory cytokine secretion as compared to CBS^+/−^ mice alone (Fig. 4d). Additionally, administration of NF-κBAI4 or NaHS in the CBS^+/−^ mice, also potently reversed NF-κB mediated inhibition of RUNX2 and osteocalcin (OCN) gene expression (Fig. 4e). Collectively, these data demonstrate that H_2_S is essential for osteogenic gene expression by suppressing histone acetylation dependent NF-κB signaling activation and inflammation (Fig. 4).

### Effect of H_2_S on Osteoclastogenesis *in vitro*

**Figure 5:**
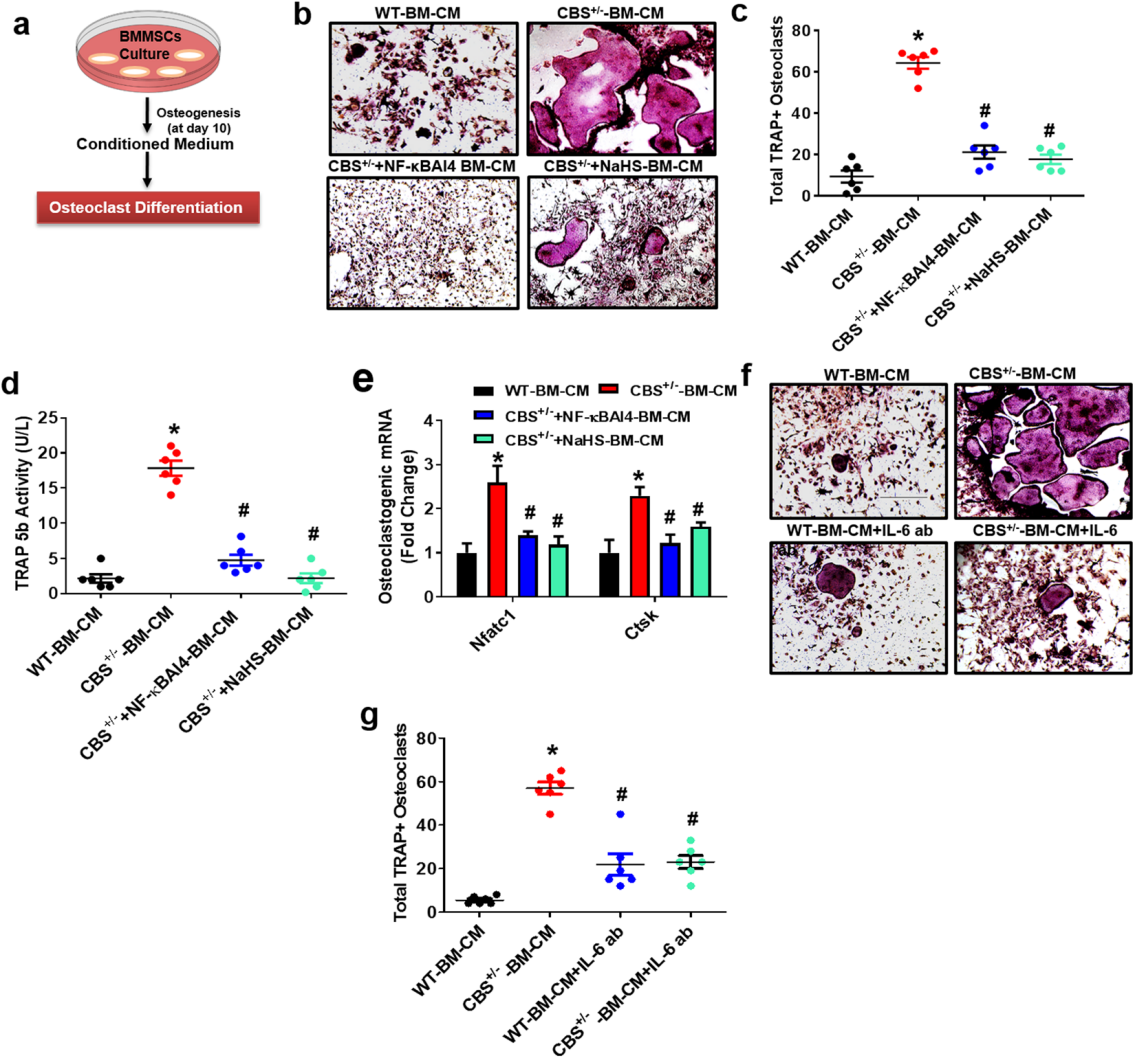
Administration of NaHS on osteoclastogenesis *in vitro*. (**a**) Experimental strategy for induction of osteoclastogenesis after treatment with BMMSCs derived conditioned medium (BM-CM). (**b**,**c**) Osteoclasts were TRAP-stained at day 5. TRAP+ osteoclasts were increased in CBS-deficient BMMSCs derived CM (CBS^+/−^ BM-CM). NaHS reversed these changes. (**d**) TRAP 5b activity was increased under CBS^+/−^ BM-CM, as assessed by ELISA. (**e**) qPCR was performed for the indicated genes: Nfatc1, Ctsk. (**f**) CM collected from CBS^+/−^ BMMSCs culture was pre-treated with the IL-6 neutralizing antibody that recognizes IL-6. Day 5 TRAP-stained mature osteoclasts. (**g**) Quantification of TRAP+ osteoclasts. Experiments were repeated three times. Data are expressed as mean ± SEM. n = 5 mice per group. *p < 0.05 compared with the WT mice, ^#^p < 0.05 compared with the CBS^+/−^ mice.

To determine whether the secretome of CBS-deficient BMMSCs contributes to bone resorption, *in vitro* osteoclast assays and plasma tartrate-resistant acid phosphatase (TRAP) activity were monitored. Bone marrow (BM) monocytes were collected from WT C57BL/6 mice and incubated under 15% conditioned medium (CM) derived from BMMSCs of experimental mice (Fig. 5a). The data revealed that CBS^+/−^ deficient BMMSCs derived CM (CBS^+/−^ BM-CM) produced more mature TRAP+ positive osteoclasts at day 5 as compared to CM from WT BMMSCs (WT-BM-CM) (Fig. 5b,c). TRAP 5b activity was also indeed increased in the osteoclast cultures incubated with CBS^+/−^-BM-CM (Fig. 5d). Interestingly, total TRAP+ osteoclasts and TRAP5b activity were normalized in CBS^+/−^ derived BMMSCs (CBS^+/−^ BM-CM) that were treated with NaHS and NF-κB Activation Inhibitor IV (NF-κBAI4) treatment (Fig. 5). In addition, we also examined the osteoclastic gene (Nuclear factor of activated T-cells, cytoplasmic 1 (Nfatc1) and Cathepsin K (Ctsk)) expression by qPCR. Expression of osteoclastic genes (Nfatc1 and Ctsk) was also increased in osteoclast cultures incubated with the CBS^+/−^ BM-CM as compared to WT-BM-CM (Fig. 5e).

We tested the efficacy of IL-6 in CM of BMMSCs, which activates osteoclastogenesis in a paracrine dependent manner. Moreover, an IL-6 neutralizing antibody that targets IL-6, was applied to the CM and we examined osteoclastogenesis progression. We found reduced osteoclastogenesis and total TRAP+ positive activity from the CM of CBS-deficient BMMSCs that were treated with the IL-6 neutralizing antibody (CBS^+/−^ CM + IL-6-Ab) as compared to CBS^+/−^ BM-CM (Fig. 5f,g). These results suggest that HHcy in BMMSCs induced the secretion of inflammatory cytokines, which eventually upregulated osteoclastogenesis. Interestingly, NaHS treatment reversed the HHcy mediated osteoclastgenesis.

### H_2_S Treatment Promotes Osteogenesis *in vitro*

**Figure 6:**
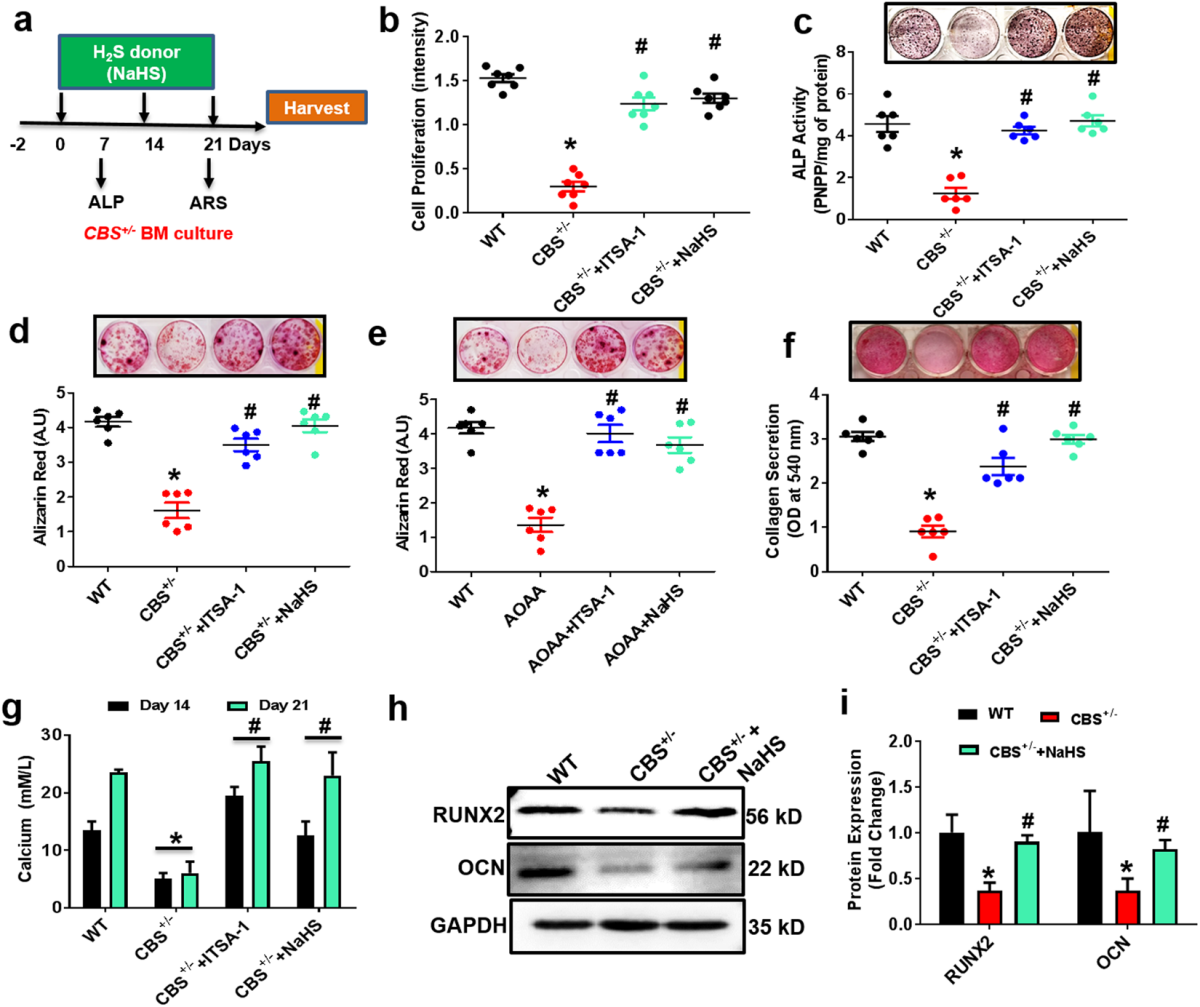
Administration of NaHS BMMSC osteogenesis *in vitro*. (**a**) *In vitro* administration of H_2_S donor (NaHS) in BMMSCs culture for 3 weeks. (**b**–**d**) Effect of NaHS on the BMMSCs osteogenesis, as assessed by MTT proliferation assay (**b**) ALP activity (**c**) and bone mineralization assay by Alizarin Red staining (ARS) (**d**). (**e**) Effect of HDAC3 activator ITSA-1 on the BMMSCs osteogenesis *in vitro*. (**f**) Collagen secretion on day 21 was studied by Sirius red assay. (**g**) Biochemical assay confirmed calcium accumulation during BMMSCs culture on day 14 and 21. (**h**,**i**) Western blot analysis of osteogenic proteins, RunX2 and OCN. Experiments were repeated three times. Data are expressed as mean ± SEM. n = 6 mice per group. *p < 0.05 compared with the WT mice, ^#^p < 0.05 compared with the CBS^+/−^ mice.

To determine whether NaHS could prevent CBS^+/−^ BMMSCs derived osteoblast dysfunction and mineralization *in vitro*, we administrated NaHS in cell culture conditions during osteogenic induction (Fig. 6a). Interestingly, data suggests that cell proliferation as well as alkaline phosphatase (ALP) activity was decreased in CBS^+/−^ derived BMMSCs culture as compared to WT. Treatment of NaHS and HDAC3 activator ITSA-1 normalized the proliferation and osteoblast ALP activity (Fig. 6b,c). After 3 weeks of osteogenic induction, Alizarin Red staining showed that NaHS or HDAC3 activator ITSA-1 treatment promoted osteogenic conversion as shown by the increase in cellular calcium nodule formation in CBS^+/−^ BMMSCs culture (Fig. 6d). When treated with the CBS inhibitor AOAA, BMMSCs showed a similar osteogenic phenotype to the one observed in CBS^+/−^ BMMSCs by decreasing bone mineralization *in vitro*. However, administration of ITSA-1 enhanced bone mineralization in AOAA treated BMMSCs culture *in vitro* (Fig. 6e). In addition, collagen secretion was improved in CBS^+/−^ BMMSCs with NaHS or HDAC3 activator ITSA-1 treatment as compared to CBS^+/−^ BMMSCs alone (Fig. 6f). Interestingly, administration of NaHS or HDAC3 activator ITSA-1 to CBS^+/−^ BMMSCs culture rescued the calcium level as compared to CBS^+/−^ BMMSCs culture (Fig. 6g). Meanwhile, Western blot results showed that the expression levels of the osteogenic markers Runx2, and OCN were significantly decreased in CBS^+/−^ derived BMMSCs as compared to WT (Fig. 6h,i). Conversely, the application of NaHS increased their induction (Fig. 6h,i). Our data support the notion that H_2_S is required for osteoblast biologic activity, including differentiation, maturation, and mineralization.

### Effect of H_2_S on Bone Formation *in vivo*

**Figure 7:**
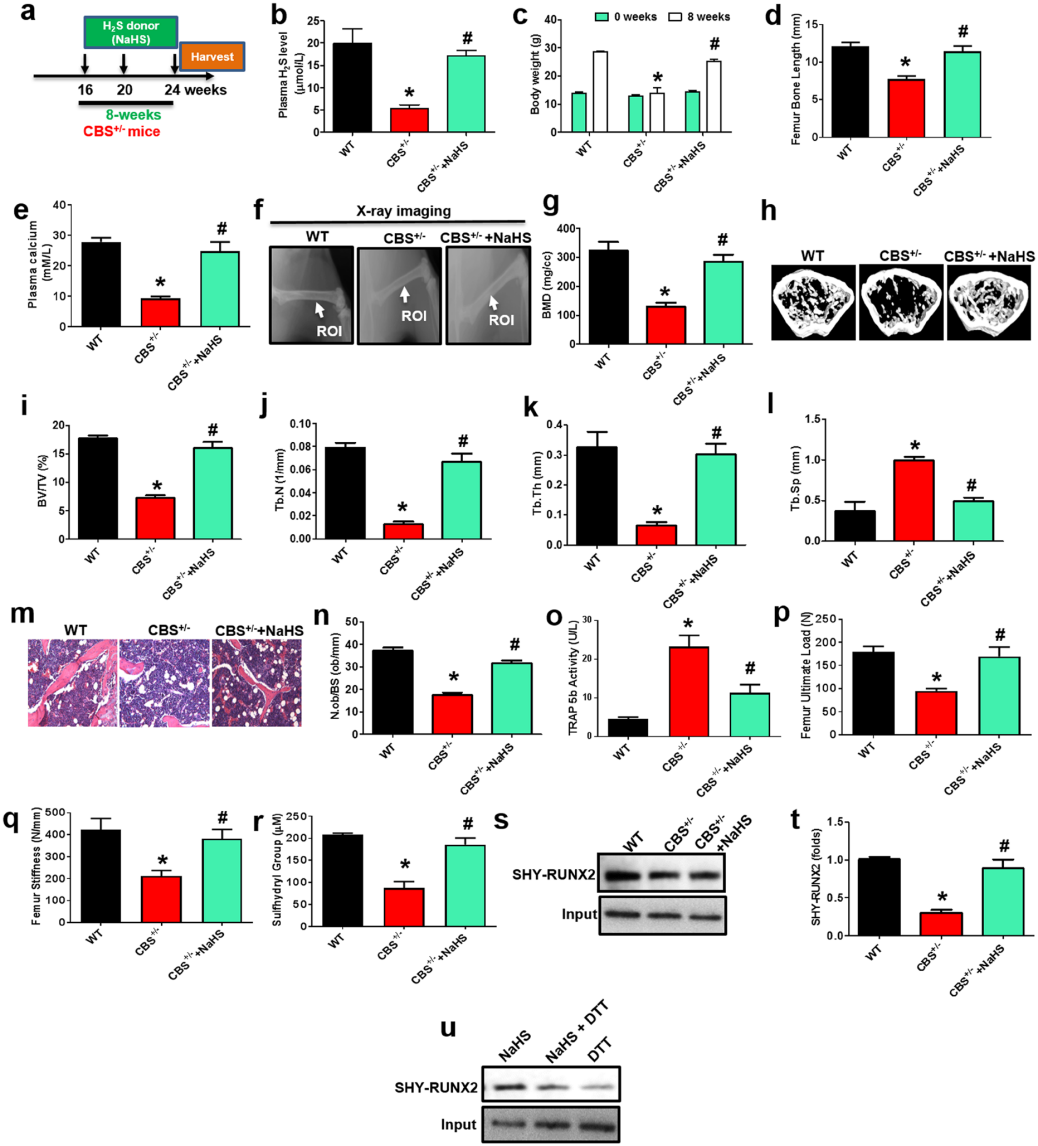
Administration of NaHS on bone formation in CBS^+/−^ mice. (**a**) NaHS was injected intraperitoneally into the experimental mice for 8 weeks. Following injections, the samples were harvested for successive experiments. (**b**) Plasma H_2_S levels were indeed increased in CBS^+/−^ mice treated with NaHS. (**c**,**d**) Both body weight and femur length were improved after NaHS injection to CBS^+/−^ mice. (**e**) In comparison with CBS^+/−^ mice, calcium levels were upregulated in CBS^+/−^ mice that were treated with NaHS, as assessed by calcium assay kit. (**f**,**g**) Representative x-ray images of the femur of experimental female mice (**f**). Arrow illustrates bone mineral density (BMD) was increased in the femur (FROI, femur region of interest) of CBS^+/−^ mice treated with NaHS (**g**). (**h**–**l**) Representative µCT cross-sectional images of distal femurs in the experimental mice. The bone phenotype parameters were observed: Bone volume per tissue volume (BV/TV) (%), trabecular number (Tb.N) (1/mm), trabecular thickness (Tb.Th.) (mm) and trabecular separation (Tb.Sp.) (mm). (**m**,**n**) Femur trabecular bone volume was increased in CBS^+/−^ mice treated with NaHS, as depicted by hematoxylin and eosin (H & E) staining. (**o**) After NaHS injection, TRAP+ activity was significantly decreased in the plasma of bone femurs. (**p**,**q**) The graph represents the biomechanical quality of the femurs: ultimate load and stiffness. NaHS reverses the HHcy induced reduced biomechanical quality. (**r**) After NaHS injection, CBS^+/−^ BMMSCs showed an increased level of free sulfhydryl groups. (**s**,**t**) RunX2 sulfhydration was significantly decreased in CBS^+/−^ mice derived BMMSCs, as assessed by immunoprecipitation with RunX2 antibody-linked biotin switch assay. (**u**) NaHS increased RunX2 sulfhydration in BMMSCs, which was removed by treatment of dithiothreitol (DTT). Data are expressed as mean ± SEM. n = 6 mice per group. *p < 0.05 compared with the wild-type (WT) mice, ^#^p < 0.05 compared with the CBS^+/−^ mice.

We next determined the *in vivo* efficacy of H_2_S treatment that mitigates BMMSCs dysfunction and bone loss in CBS-deficient mice, where NaHS was administrated (i.p. injections at 10 mg/kg/day) into the 16-week-old female CBS^+/−^ mice for 8 weeks. The detailed experimental design was presented as shown in Fig. 7a. Ultimately, plasma levels of H_2_S were indeed increased in CBS^+/−^ mice as shown in Fig. 7b. We also measured total body weight in the experimental mice at both 16-weeks and 24-weeks of age respectively (as 0 and 8-weeks) (Fig. 7c). H_2_S rescued the CBS-deficiency reduced body weight and femur length (Fig. 7c,d). Calcium is an important element for bone formation^31^, therefore we measured the calcium level in plasma of the experimental mice. As expected, administration of NaHS, elevated the plasma level of calcium in CBS^+/−^ mice (Fig. 7e). To investigate the effects of NaHS on the bone mass phenotype, radiographs of femur bones were taken by a FX PRO Imaging System. The results showed that reduced quality of the femur, as evidenced by bone densitometry measurements, created changes in bone density similar to what was observed in CBS^+/−^ mice. However, the effect was reversed with administration of NaHS (Fig. 7f,g). Representative micro-computed tomography (µCT) images of experimental femur bones demonstrated that CBS^+/−^ mice had lower trabecular bone volume per tissue volume (BV/TV), trabecular number (Tb.N), trabecular thickness (Tb.Th) and increased trabecular separation (Tb.Sp) (Fig. 7h,i). 2D histological evaluation of the mouse distal femur also confirmed the decrease in trabecular bone volume in CBS^+/−^ mice, indicating osteoporosis. However, administration of NaHS reversed these changes (Fig. 7m,n).

CBS^+/−^ mice showed a significant increase in TRAP+ activity in the plasma of their femurs (Fig. 7o), suggesting that decreased H_2_S levels may be associated with accelerated osteoclast activity (Fig. 5). Moreover, CBS^+/−^ mice showed a significant decrease in ultimate load and stiffness of femurs compared with WT control (Fig. 7p,q). However, NaHS supplementation reversed the H_2_S deficiency induced detrimental effects in the bone of CBS^+/−^ mice. Collectively, these data indicate that H_2_S is required for normal bone homeostasis, and potentially mitigates CBS-deficiency induced osteoporosis. Further, these observations suggested that H_2_S deficiency induced the generation of ROS in the BMMSCs, potentiating osteoporotic bone loss.

### Effect of H_2_S on RUNX2 Sulfhydration

To further understand the mechanistic study of H_2_S driven bone formation, we sought to examine the post-translational modification of RUNX2 transcription. Protein sulfhydration at cysteine sites is an important regulatory mechanism of H_2_S in the cellular system^42^. Sulfhydration (SHY) is a process whereby H_2_S modifies specific Cys residues (−SH) in proteins through persulfide (-SSH) bonds^31^. Protein sulfhydration is involved in various physiological functions such as cell differentiation, endoplasmic reticulum stress, mitochondrial biogenesis, oxidative stress and inflammation^31,43,44^. In the present study, we assessed the free sulfhydryl groups (−SH) in the BMMSCs using Ellman’s method. The data suggests that the free sulfhydryl groups were decreased in CBS^+/−^ BMMSCs as compared to WT. However, NaHS treatment in BMMSCs increased the number of free sulfhydryl groups (Fig. 7r). Using a modified biotin switch assay, we studied whether H_2_S affects sulfhydration of RUNX2. The results showed that CBS^+/−^ mice had lower RUNX2 sulfhydration activity in BMMSCs as compared to WT (Fig. 7s,t). However, administration of NaHS induced endogenous RUNX2 sulfhydration in CBS^+/−^ BMMSCs. Additionally, we also tested whether NaHS induced endogenous RUNX2 sulfhydration in BMMSCs, which was removed by the desulfhydration reagent, dithiothreitol (DTT) (Fig. 7u). These data suggest that RUNX2 transcription factor was sulfhydrated after H_2_S treatment and mediated osteogenesis.

## Discussion

In this study, we elucidate the essential functions of H_2_S in HHcy reduced bone formation in CBS^+/−^ mice. Mice in which CBS-deficiency in BMMSCs also exhibited a decreased level of endogenous H_2_S production demonstrated: exaggerated oxidative damage through HHcy; accelerated bone resorption; severely reduced bone mineral density; and reduced trabecular bone volume fraction. Clinical studies have also confirmed that CBS-deficient patients experience a wide range of destructive phenotypes, including cognitive impairment, premature atherosclerosis, dislocated lenses, thrombosis, vascular complications and osteoporosis^45^. Our results demonstrate that CBS mediated H_2_S controls intercellular communication between BMMSCs and BM-derived osteoclasts required for bone development. In particular, we observed that CBS deficiency in BMMSCs activates inflammation in the bone milieu by upregulating many inflammatory cytokines that appear to be caused by osteoclastic bone loss during bone formation. *In vitro* cell culture studies revealed that increased osteoclast activity was due to high Hcy content via oxidative damage^10,13,46^. In contrast with the previous study, our data suggest that osteoclast activity was significantly higher via HHcy mediated inflammatory cytokines, IL-6 and TNF-α in CBS deficient mice. Interestingly, cytokines such as IL-6 are classic inflammatory factors that mediate potent biological effects by regulating cell-cell communication in the HHcy condition. Inflammatory factors produced by BMMSCs can diffuse across trabecular bone areas and mediate osteoclast activation and differentiation, thereby initiating bone resorption. However, the epigenetic alternation of inflammatory signaling and subsequent deterioration of the trabecular bone mass phenotype during HHcy in CBS deficient mice has not yet been elucidated.

The previous study also reported that HDAC3 is required for maintenance of trabecular bone mass during skeletal development. HDAC3 conditional knockout (CKO) mice exhibited trabecular bone loss, decreased bone mineralization and material properties, as well as frequent fractures^47^. Our result demonstrates, for the first time, that HHcy is an intriguing factor that negatively inhibits HDAC3 activity in CBS^+/−^ mice. Furthermore, we propose that HHcy-mediated suppression of HDAC3 activity is sufficient to enhance inflammatory cytokine transcriptional activation in BMMSCs. Our data show that CBS deficiency causes HDAC3 inhibition induced proinflammatory cytokine signaling by hyperacetylating histones and NF-κB (p65) in BMMSCs. The previous report explained the robust NF-κB activation and expression of cytokines observed in HDAC3-deficient mice of T regulatory lymphocytes and chondrocytes^16,48^. Consistent with the earlier report, our work found that NF-κB acetylation and inflammatory cytokines were robustly increased in BMMSCs, in part; by inducing acetylation at lysine 27 of H3 (H3K27 ac) in CBS^+/−^ mice. Both IL-6 and TNF-α were highly induced and mediated inflammatory paracrine signaling. Thereby osteoclastogenesis was increased via IL-6 stimulation. A blockade of NF-κB (p65) signaling by the specific inhibitor, NF-κBAI4, suppressed inflammatory cytokine expression. Additionally, prevention of HDAC3 inhibition by administrating HDAC activator, ITSA-1, balanced deacetylation activity and suppressed IL-6 and TNF-α expression. In summary, our study provides the mechanistic role of HHcy mediated inflammatory transcriptional gene regulation via deregulation of HDAC activity and subsequent acetylation of histone and NF-κB.

Cystathionine β-synthase (CBS) is the first enzyme that catalases homocysteine to cystathionine and also produces H_2_S during transsulfuration of methionine metabolism. Mutation of CBS leads to advancement of the HHcy phenotype and decreased bioavailability of H_2_S^49^. Previously we also demonstrated that CBS expression was downregulated in excess high methionine diet (HMD) fed mice, leading to decreased H_2_S biosynthesis by elevating the intracellular pool of Hcy in BMMSCs of mice^41^. Administration of H_2_S restores the physiological level of H_2_S and also decreases the Hcy level in mice. However, CBS-deficiency induced metabolic bone loss is not well studied yet. As the H_2_S level was ablated in the *CBS*^+/−^ mice, we embarked to understand whether, H_2_S supplementation could restore the CBS-deficiency induced bone catabolism *in vivo*. In the present study, we showed that NaHS administration to CBS^+/−^ mice rescued the HHcy reduced BMMSCs mediated osteogenesis by upregulating ALP activity, calcium accumulation and collagen secretion in *in vitro* culture conditions. Interestingly, we found that HDAC activity is overall increased upon administration of NaHS in the CBS-deficient condition. Also, treatment with HDAC activator ITSA-1 to culture conditions, rescued the CBS-deficient ablated osteoblast differentiation and mineralization. These data suggest that H_2_S deficiency in the bone microenvironment of CBS^+/−^ mice plays a critical role in HHcy mediated dysfunctional osteogenesis through HDAC inhibition in BMMSCs.

It is well known that sulfur-containing amino acids are closely associated with bone homeostasis^50^. H_2_S is a metabolic production of homocysteine by CBS, CSE or 3-mercaptopyruvate sulfurtransferase (MPST) enzyme activity^31,42,43^. In this study, we demonstrated that the role of the CBS-H_2_S system in BMMSCs regulates bone formation via protein sulfhydration. Importantly, the number of free sulfhydryl groups was diminished in CBS-deficient BMMSCs. However, administration of DTT also increased the number of free sulfhydryl groups. The recent study demonstrated that NaHS induced endogenous RUNX2 sulfhydration at C123 and C132 sites in cultured osteoblasts using CSE-H_2_S system. Here, we demonstrated RUNX2 sulfhydration in CBS^+/−^ BMMSCs and NaHS administration in CBS^+/−^ BMMSCs was also verified. Using a biotin switch assay, we concluded that the NaHS induced RUNX2 protein sulfhydration was indeed increased in CBS^+/−^ BMMSCs. However, the protein sulfhydration level was diminished in CBS-deficient BMMSCs and in DTT only treated BMMSCs, suggesting that H_2_S at the physiological level plays an important role in regulating the normal function of RUNX2 activity in BMMSCs. This study demonstrates that bone formation was unfavorably affected by HHcy in CBS-deficiency. Future research will need to be performed on the osteoclast phenotype and BMMSCs functions in CBS^+/−^ mice to fully understand the pathobiology of epigenetic deregulation and inflammation in destructive diseases such as osteoporosis.

**Figure 8:**
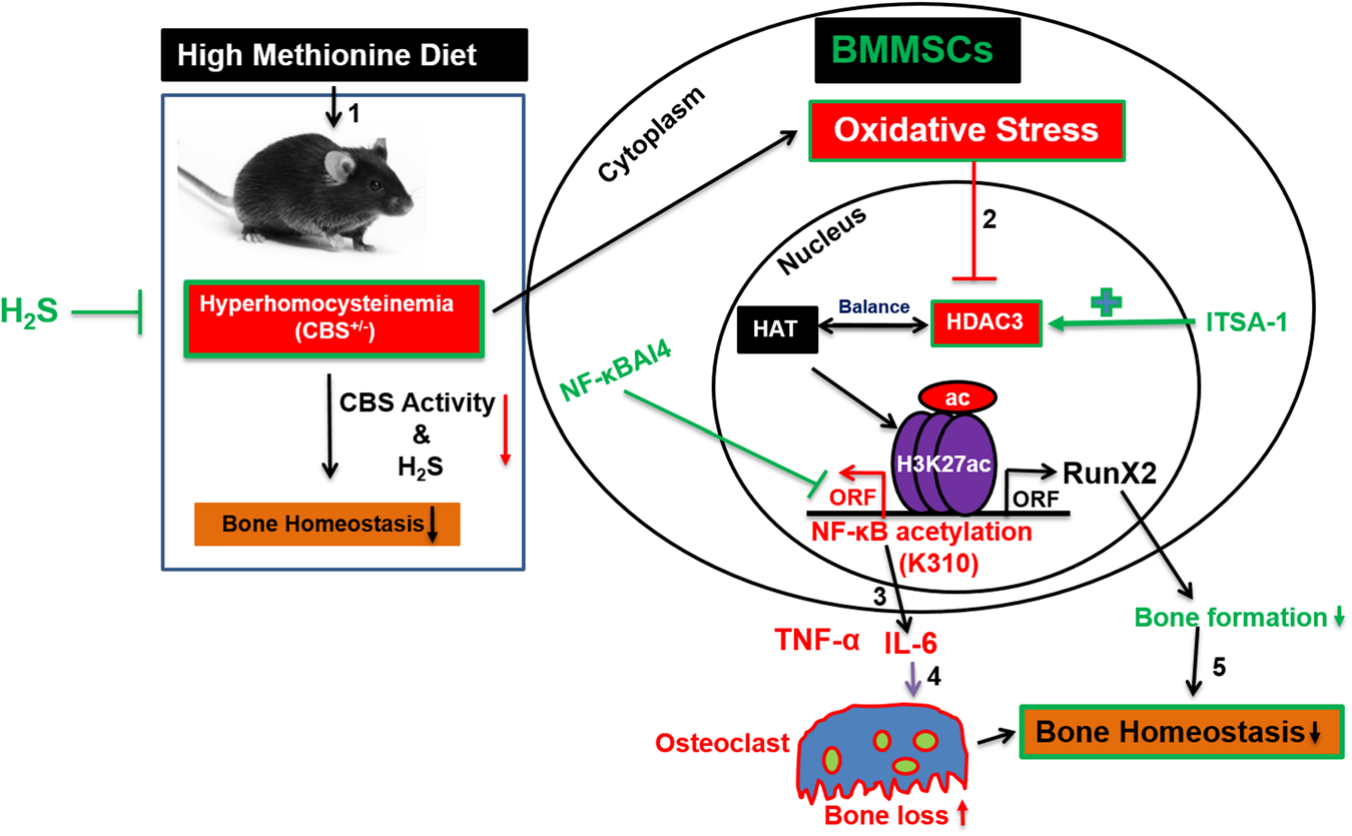
Proposed mechanism for the role of H_2_S on HHcy induced bone loss in CBS^+/−^ mice. First, the HHcy condition is induced in CBS^+/−^ mice by an alteration of CBS enzyme activity and decreased H_2_S production. Second, the HHcy condition further inhibits HDAC activity through oxidative stress. However, administration of NaHS (H_2_S donor) or specific HDAC activator (ITSA-1) reverses these effects in CBS^+/−^ mice. Third, inhibition of HDAC3 further epigenetically regulates histone acetylation and inflammatory gene expression. Fourth, exaggerated inflammatory cytokines expression enhances osteoclastogenesis and bone loss. Fifth, HHcy mediated decreased RunX2 sulfhydration, is reversed by NaHS, thereby bone homeostasis is maintained.

In summary, H_2_S plays a crucial role in regulating BMMSCs transcriptome, by modifying the chromatin landscape in the CBS-deficient mice model and plays a potential role in bone formation. Loss of CBS function in BMMSCs enhances HHcy phenotypes, upregulates inflammatory signaling, alters osteogenesis by increasing osteoclastogenesis, ultimately causing trabecular bone loss. Furthermore, our study also explains the molecular mechanism of H_2_S that balances the key epigenetic regulator HDAC3, as well as inflammatory NF-κB signaling in the HHcy condition that arises in BMMSCs of CBS-deficient mice. Therefore, our data strongly suggests that H_2_S could be a potential therapeutic agent for treating osteoporosis arisen due to CBS-deficiency through epigenetic modulation of osteogenesis (Fig. 8).

## Materials and Methods

### Animals and Experimental Design

Female C57BL/6 J and 129P2-*Cbs*^*tm1Unc*^/J mice were purchased from the Jackson Laboratory (Harbor, ME). The animal procedures were carefully reviewed and approved by the Institutional Animal Care and Use Committee of the University of Louisville and followed the animal care and guidelines of the National Institutes of Health. The animals were fed standard chow and water *ad libitum*. To develop the HHcy condition, female (16–week-old) CBS^+/−^ mice were fed with a methionine enriched (1.2%), low folate (0.08 mg/Kg), low vitamin B6 (0.01 mg/Kg) and B12 (10.4 μg/Kg) diet (Harlan Laboratories, Cat No.TD.97345) for 8 weeks. We measured total body weight in the experimental mice at both 16-weeks (considered as 0-weeks) and 24-weeks (as 8-weeks) of age. To study the beneficial effects of H_2_S on bone homeostasis in CBS^+/−^ mice, sodium hydrogen sulfide (NaHS) was intraperitoneally (i.p) injected for the 8 week period. The mouse groups were as follows:

- Wild-type C57BJ/L6 mice (WT)
- CBS^+/−^ mice fed with HMD (CBS^+/−^ +HMD, as HHcy mice model)
- NaHS-supplemented CBS^+/−^ mice (HHcy + NaHS)
- NF-κB Activation Inhibitor IV - supplemented CBS^+/−^ mice (CBS^+/−^ +NF-κB IV)
- ITSA-1 (HDAC activator)- supplemented CBS^+/−^ mice (CBS^+/−^ +ITSA-1)

### Drug Preparation and Administration

#### In vivo

NaHS (H_2_S precursor), NF-κB IV (NF-κB activation inhibitor IV), ITSA-1 (HDAC activator) and NAC (anti-oxidant), were dissolved in 0.9% normal saline. CBS^+/−^ mice were treated with NaHS (10 mg/kg/day) given through intraperitoneal route for a period of 8 weeks. NF-κB IV (2 mg/kg body weight), ITSA-1 (0.5 mg/kg body weight) treatments were given 3 times/week through intraperitoneal route for a period of 8 weeks. Animals of the control group received normal saline through i.p injections. After 24 hours of the last NaHS treatment or its vehicle injection, samples were collected for biochemical, molecular and immunohistochemical analyses.

#### In vitro

The BMMSCs used for *in vitro* study have been described in previous studies^31^ and were given the following treatments: (1) WT-BMMSCs, (2) CBS^+/−^-BMMSCs, (3) CBS^+/−^-BMMSCs + ITSA-1 (12.5 *μM*), (4) CBS^+/−^-BMMSCs + NaHS (100 *μM*). Cells were given treatment for 3-weeks and were then harvested for further experimentation.

### Isolation of Mouse BMMSCs and its Characterization

Mouse BMMSCs were isolated as per previously described with some modification^31^. Mouse bone marrow cells were flushed out from the bone cavities of femurs of 18-week old female mice by centrifugation (3000 rpm for 10 min at room temperature) and collected with 2% heat-inactivated fetal bovine serum (FBS; ATCC) in alpha minimum essential medium (α-MEM; Invitrogen). The cells were washed thrice with PBS and suspended in α-MEM 15% FBS. Cells were seeded at 1.6 × 10^6^ into 6-well culture plates (Corning) and initially incubated for 24 hr at 37 °C in a 5% CO_2_ incubation chamber. The non-adherent cells were washed twice with phosphate-buffered saline (PBS). The adherent cells were further cultured for 14 days. The BMMSCs were cultured with α-MEM supplemented with 15% FBS, 2 mM L-glutamine (Invitrogen), 100 U/ml penicillin (Invitrogen), 100 mg/ml streptomycin (Invitrogen) and 50 mg/ml Amphotericin B (Invitrogen). The BMMSCs were characterized by flow cytometry analysis with specific markers such as CD73 and CD44 (Biolegends, San Diego, CA).

### *In Vitro* Mineralization Staining Assay

*In vitro* mineralization was performed as previously described^41^. For *in vitro* ARS assay, BMMSCs were cultured under osteogenic induction medium (α-MEM + 15% FBS supplemented with 2 mM β -glycerophosphate, 100 nM dexamethasone and 50 μg/mL ascorbic acid) in 12-well culture plates with or without NaHS for 21 days. Cells were then washed with PBS, fixed with 70% ethanol for 35 minutes and stained with 2% Alizarin Red-S (AR-S) stain (Sigma Aldrich) with pH 4.2 for 25 minutes at room temperature. Following staining, all images were taken on a phase contrast microscope. Briefly, absorbance was read at 510 nm calorimetrically for quantification of bone mineralized nodules.

### Alkaline Phosphatase (ALP) Assay

ALP activity and staining was carried out to study the osteogenic potential of BMMSCs, as described previously^41^. Briefly, BMMSCs were cultured under osteogenic induction medium at a density of 5.2 × 10^4^ cells/well in 12-well culture plates. After 7 days of osteogenic induction, the cells were fixed with 70% ethanol for 30 minutes and stained for ALP (Sigma) at room temperature and the images were then photographed. Also, enzymatic activity was measured by spectrophotometric detection of p-nitrophenol product at 405 nm and expressed PNPP/mg of protein.

### Osteoclast Assay

Osteoclasts were cultured as previously described^51^. BM cells were collected with α-MEM (15% FBS) from the cavity of femurs and tibias of 18–week-old mice and BM cells were plated at 5 × 10^5^ cells/well in 24-well culture plates under α-MEM, then treated with RANKL (30 ng/mL) and M-CSF (10 ng/mL) for 5 days. The media was changed every alternative day. After 5 days of culture, cells were fixed and stained with tartrate-resistant acid phosphatase (TRAP) using a Leukocyte Acid Phosphatase kit (Sigma). TRAP-positive cells were photographed for six fields and the means of the cell numbers were calculated. Similarly, osteoclast activity was measured by TRAP5b activity in plasma, using an ELISA kit from MyBioSource as per the manufacturer’s instructions.

### Measurement of IL-6 and TNF-α by ELISA

ELISA assay was used to determine the amount of IL-6 and TNF-α in the BM plasma of different experimental mice. BM plasma-derived IL-6 and TNF-α were measured using a commercial mouse IL-6 (ab100712) and TNF-α (ab100747) sandwich ELISA kit from Abcam, as per the manufacturer’s instructions.

### Homocysteine Measurement Assay

This assay was used to measure the total homocysteine content in the plasma using a Homocysteine assay kit from Crystal Chem as per the manufacturer’s instructions.

### Measurement of Glutathione Peroxidase (GPx) Activity

This assay is used to measure all of the glutathione-dependent peroxidases in plasma and BMMSCs lysate using a Glutathione Peroxidase assay kit from Abcam as per the manufacturer’s instructions.

### Hydrogen Sulfide (H_2_S) Measurement Assay

The endogenous level of H_2_S was measured using BM plasma and culture medium, as previously described^41^. Then the bone femurs of experimental mice were homogenized in ice-cold 50 mmol/L potassium phosphate buffer, pH 8.0 and the homogenate was centrifuged (10,000 g; 12 minutes; 4 °C). The collected supernatant (75 µL) was mixed with 0.25 mL Zn acetate (1%) and 0.45 mL water and was then incubated for 30 minutes at room temperature. Following incubation, TCA (10%; 0.25 mL) was then added to the mixture and centrifuged (12,000 g; 25 minutes; 4 °C). The collected supernatant was mixed with N,N-dimethyl-p-phenylenediamine sulfate (20 mmol/L; 133 µL) in 7.2 mol/L HCl and FeCl3 (30 mmol/L; 133 µL) in 1.2 mol/L HCl. After 30 minutes of incubation, absorbance was measured at 670 nm with a microplate reader. A similar procedure was also followed to measure H_2_S in the culture medium. The standard curve of H_2_S was generated by using varying concentrations of NaHS solution (0 to 320 µmol/L NaHS).

### Intracellular ROS Analysis by Flow Cytometry

Briefly, cultured BMMSCs (5 × 10^4^ cells) were stained with 5 mM of dichlorodihydrofluorescein diacetate (CM-H2DCFDA, Invitrogen, Carlsbad, CA, USA) for 30 minutes at 37 °C. The stained cells were washed twice with 1X PBS. Stained cells were then analyzed using flow cytometer (BD Accuri™ C6 Plus). The average intensity of ROS fluorescence was analyzed using the BD Accuri C6 Software.

### Lipid Peroxidation Assay

This assay was used to measure the cellular total lipid peroxide level in the form of malondialdehyde (MDA) content in the bone plasma using a Lipid Peroxidation (MDA) assay kit from Abcam as per the manufacturer’s instructions.

### Western Blot Analysis

The femur bone extracts (50 μg) were prepared with RIPA lysis buffer and loaded on a sodium dodecyl sulfate–polyacrylamide gel electrophoresis (SDS-PAGE), and run at a constant 110 volts. Following separation of proteins, the gels were transferred to polyvinylidene difluoride membranes using an electrotransfer apparatus (Bio-Rad) for 3–4 hours. Following transfer, membranes were blocked with 5% non-fat dry milk in TBS-T solution for 45 mins. Then, membranes were incubated with the desired primary antibody for 3 hours at 4 °C. After washing with TBS-T 3-times each for 7 mins, the membranes were further probed with a secondary antibody [horseradish peroxidase-conjugated] for 2 hours at room temperature. The membranes were then developed with ECL Western blotting detection system (GE Healthcare, Piscataway, NJ, USA) and imaged on gel documentation system (Bio-Rad). Band density was normalized with respective housekeeping gene control using Image Lab densitometry software (Bio-Rad).

### Gene Expression by Quantitative Real-Time PCR (qRT-PCR)

The quantitative gene expression profile of mRNA transcript levels was performed using qRT-PCR. Total RNA was isolated with TRIzol® reagent (Invitrogen, Grand Island, NY, USA) from the whole femur bone tissue as per the manufacturer’s instructions. Quantification of the total RNA was assessed using Nanodrop-1000 (Thermo Scientific, Waltham, MA, USA). Total RNA (2 μg) was used for reverse-transcription to obtain cDNA, as per previously described^52^. Briefly, cDNA was prepared in a thermocycler (Bio-Rad) with the following cycle: 42 °C for 55 min, 70 °C for 18 min and 4 °C at the end. The qRT-PCR was performed for target genes in a final reaction volume of 20 μl by using LightCycler Instrument, Roche. Relative quantification of PCR products was calculated according to the loading control mRNA expression of GAPDH. The list of quantitative gene expression primers used in this study is shown in Table 1.

**Table 1:** Sequences of PCR primers used for real time quantitative PCR and ChIP assay PCR.

Gene	Primer Sequences (5′→3′)
Mouse Runx2	FP: TTTAGGGCGCATTCCTCATC RP: TGTCCTTGTGGATTGAAAGGAC
Mouse Osteocalcin	FP:GCGCTCTGTCTCTCTGACCT RP: ACCTTATTGCCCTCCTGCTT
Mouse IL-6	FP:CCTCTGGTCATCTGGAGTACC RP:ACTCCTTCTGTGACTCGAGC
Mouse TNF-α	FP:ATGAGCACAGAAAGCATCA RP:AGTAGACAGAAGAGCGTAGT
Mouse Nfatc1	FP:GAGACAGACATCCGGAGGAGA RP:GTGGGATGTGAACACGGAAGA
Mouse Cathepsin K	FP:GGATGAAATCTCTCGCGTTT RP:GGTTATGGGCAGAGATTGCTT
Mouse CBS	FP:AGGGCTATCGCTGCATTATCTGA RP:AGCTTCCACCACATAGCAGTCCTT
Mouse GAPDH	FP: TGCACCACCAACTGCTTGC RP: GGCATGGACTGTAGTCAGAG
**Gene**	**ChIP assay Primer sequences (5′→3′)**
Mouse IL-6	FP:CACTTCACAAGTCGGAGGCT RP:AATGAATGGACGCCCAGACT
Mouse TNF-α	FP:CAACAGCTCAAGTCTTCCCTGAT RP:CCCCTGGGATGCCTAGAAGT
Mouse Runx2	FP:TGGTAGGCAGTCCACTTTAC RP:GGCTGGTAGTGACTGCAGAG
Mouse Osteocalcin	FP:CCAGGCATCTGGAGCTAAT RP:ATAAGACAGCAGGCTGAGATG

### HDAC Activity Assay

From whole femur bone homogenate, nuclear extract was isolated using EpiQuik Nuclear Extraction Kit (Base Catalog # OP-0002, Epigentek, Farmingdale, NY). The HDAC activity assay was performed using EpiQuick HDAC Activity/Inhibition assay kit (Base Catalog # P-4001, Epigenetek) according to the manufacturer’s instructions.

### Global Acetyl Histone H3K27 Activity Assay

The histone extract was isolated from whole femur bone homogenate using EpiQuik Total Histone Extraction Kit (Base Catalog # OP-0006, Epigentek, Farmingdale, NY). Quantification of histone H3 modification was performed using EpiQuik Global Acetyl Histone H3K27 Quantification Kit (Base Catalog # P-4059, Epigentek) according to the manufacturer’s instructions.

### Generation of CBS KO Cell Lines Using CRISPR/Cas9 Gene-editing Technology

CBS KO BMMSCs were generated by using CRISPR/Cas9 system as previously described^53^. Briefly, BMMSCs were transfected with CRISPR/Cas9 KO Plasmid (Santacruz Biotechnology, sc-400877, CA, USA), by using UltraCruz® Transfection Reagent (Santacruz Biotechnology, sc-395739, CA, USA). Then cells were incubated for 48-hours at 37 °C. After incubation, successful transfection of CRISPR/Cas9 KO Plasmid was visually confirmed by detection of the green fluorescent protein (GFP) via confocal microscopy.

### Chromatin Immunoprecipitation Assay (ChIP)

Chromatin preparation in whole bone homogenate was carried out using ChromaFlash Chromatin Extraction Kit (Base Catalog # P-2001 Epigentek, Farmingdale, NY, USA) as described by the manufacturer’s instructions. Chromatin immunoprecipitation assay was performed as per previously described^41^. Briefly, the immunoprecipitated DNA (through anti-H3K27ac antibody) was amplified by PCR reaction using GoTaq® Hot Start Green Master Mix (Promega, USA) and PCR was performed by using S1000™ Thermal Cycler-BIORAD. Primers were designed for the targeted gene promoter. The primer pairs were designed to amplify sequences surroundings, which predicted H3K27ac binding sites at the target gene locus, as described in Table 1.

### Luciferase Reporter Assay

The NF-κB activity in cultured cells was tested using luciferase reporter assays as previously described^54^. To study the activity of NF-κB in BMMSCs, we performed luciferase reporter assays. Mouse BMMSCs cultures were transfected with 1 μg of the pGL4.32[luc2P/NF-κB-RE/Hygro] vector encoding the firefly luciferase reporter gene (luc2P) driven by five copies of an NF-κB enhancer element (Promega) and incubated for 48- hours at 37 °C. Transfection experiments were performed with Lipofectamine™ LTX Reagent (Invitrogen) according to the supplier’s protocol. NF-κB activation has been known to stimulate the expression of luciferase encoded in the pGL4.32[luc2P/NF-κB-RE/Hygro] vector. Cells were collected, and luciferase activity was measured using a Dual-Luciferase Reporter Assay system (Promega) and a GloMax-Multi + Detection System (Promega). Each experiment was independently repeated three times. The pGL4.27[luc2P/minP/Hygro] encoding the luc2P only (Promega) was used as the control vector.

### Sulfhydryl Groups Measured by Ellman’s Test

The free (reduced) sulfhydryl groups (-SH) in mouse plasma were measured by Ellman’s Test as previously described^31^. Briefly, 4 mg of Ellman’s Reagent (5,5′-dithio-bis-[2-nitrobenzoic acid] DTNB) was dissolved in 1 ml of reaction buffer (0.1 M sodium phosphate, pH 8.0, containing 1 mM EDTA). DTNB in the solution was reacted with a free sulfhydryl group to yield a mixed disulfide and 2-nitro-5-thiobenzoic acid (TNB). A set of test tubes was prepared, each containing 100 μl of Ellman’s reagent solution and 5 ml of reaction buffer. To analyze the sulfhydryl groups in living cells/plasma, 400 μl of Ellman’s reagent solution was directly added into each well of the culture plate and incubated at 37 °C for 25 minutes. Then the solution was collected and absorbance measured at 412 nm. Cysteine hydrochloride monohydrate was used as a reference standard.

### Immunoprecipitation Linked Biotin Switch Assay

The protein sulfhydration assay was performed as described with modifications^55^. Briefly, cultured BMMSCs were treated with H_2_S or dithiothreitol (DTT) for 7 days. Cells were homogenized in RIPA lysis buffer (Sigma-Aldrich), and centrifuged at 14,000·g (4 °C) for 15 min. The supernatant was collected and protein was quantified by a bicinchoninic acid assay. The primary anti-RUNX2 antibody (2lg) was added to the protein lysis (1 mg/mL) material containing protein A beads. After incubation overnight at 4 °C, the beads were washed and blocked with 250 mM Hepes-NaOH (pH 7.7), 1 mM EDTA and 0.1 mM neocuproine (HEN) buffer (containing 2.5% sodium dodecyl sulfate and 20 mM methyl methanethiosulfonate [MMTS]) at 50 °C for 20 min. MMTS was then removed by precipitating proteins with acetone at −20 °C for 20 min. After acetone removal, the proteins were resuspended in HEN buffer (containing 1% SDS) and 4 mM biotin-HPDP was added for incubation for 4 h at room temperature. Biotinylated protein was pulled down by streptavidin magnet beads, eluted by SDS-PAGE loading buffer and was then after subjected to Western blot analysis.

### Dynamic Histomorphometric Analysis

The femurs of experimental mice were collected and fixed in 4% para-formaldehyde overnight, then decalcified in 14% ethylenediamine tetraacetic acid (EDTA) for 10 days. The femurs were then embedded in paraffin wax. The samples were sectioned into 4 µm slices using a Cryostat Microtome LS-6150. The slices were stained with H&E solutions according to the manufacturer’s instructions and the photographic images were obtained using a phase-contrast microscope (Leica DM4000B, Germany) with 10× magnification. Seven representative images were analyzed by using NIH Image J software. The results were shown as the percentage of trabecular bone per total bone area.

### 3-point Bending Test of Bone

Bone femurs were dissected from the experimental mice and fixed in 10% neutral buffered formalin overnight and stored in −80 °C until usage. The samples were used for mechanical testing by a 3-point bending test. All bone samples were tested under a load applied at a constant rate of 20 mm/min. This experimental test predominantly measured cortical bone strength parameters, such as maximum load (ultimate strength) and stiffness (elastic deformation) by using Bone Strength Tester TK-252C (Muromachi Kikai Co. Ltd., Tokyo, Japan).

### Micro–computed tomography (µCT) Analysis of Bone

Micro-CT analyses of excised femur bone samples were carried out using the SkyScan 1076 μ-CT scanner (Aartselaar, Belgium). The bone samples were fixed in 4% para-formaldehyde overnight and stored in a 70% ethanol solution kept in −80 °C until usage. Bones were scanned using the SkyScan 1076 μ-CT scanner. After scanning, 3D-reconstructions of the bones were carried out by SkyScan Nrecon software. Following reconstruction, the trabecular bone volumes of the distal femurs were segmented by drawing ellipsoid contours with the CT analyzer software and propogating the volume through the image stack. The trabecular bone volume/tissue volume BV/TV (%), trabecular number (Tb.N) (1/mm), trabecular separation (Tb.Sp) (mm) and trabecular thickness (Tb.Th) (mm) were obtained following data analysis.

### Statistical Analysis

Data analyses and graphical presentations were performed using GraphPad Prism, version 5.00 (GraphPad Software, Inc., La Jolla, CA). Data are represented as mean values ± standard error mean (SEM). The experimental groups were compared by one-way analysis of variance (ANOVA) in combination with Tukey’s multiple comparison test. The significance of differences between groups was determined using two-tailed, unpaired Student’s t-test. P-values of less than 0.05 were considered statistically significant.

## Electronic supplementary material

Supplementary file

## Data Availability

All data generated or analyzed during this study are included in this published article.
