# Supplementary material for: Hydrogen Sulfide Promotes Bone Homeostasis by Balancing Inflammatory Cytokine Signaling in CBS-Deficient Mice through an Epigenetic Mechanism
Source: Sci Rep. 2018 Oct 15;8:15226. doi: 10.1038/s41598-018-33149-9 (PMC6189133; doi:10.1038/s41598-018-33149-9)
Supplement: Supplementary file 1 — Supplementary file [file 41598_2018_33149_MOESM1_ESM.pdf]

Supplementary information

(Full length blots/gels for the main figures in the paper)

Hydrogen sulfide maintains bone homeostasis by balancing inflammatory cytokine signaling in CBS-deficient mice through an epigenetic mechanism

Jyotirmaya Behera[1], Kimberly E. Kelly[1], Michael J. Voor[2, 3], Naira Metreveli[1], Suresh C. Tyagi[1], Neetu Tyagi[1]*

[1]Bone Biology Laboratory, Department of Physiology, School of Medicine, University of Louisville, Louisville, KY 40202
[2]Department of Orthopaedic Surgery, School of Medicine, University of Louisville, Louisville, KY 40202 USA
[3]Department of Bioengineering, Speed School of Engineering, University of Louisville, Louisville, KY 40292 USA

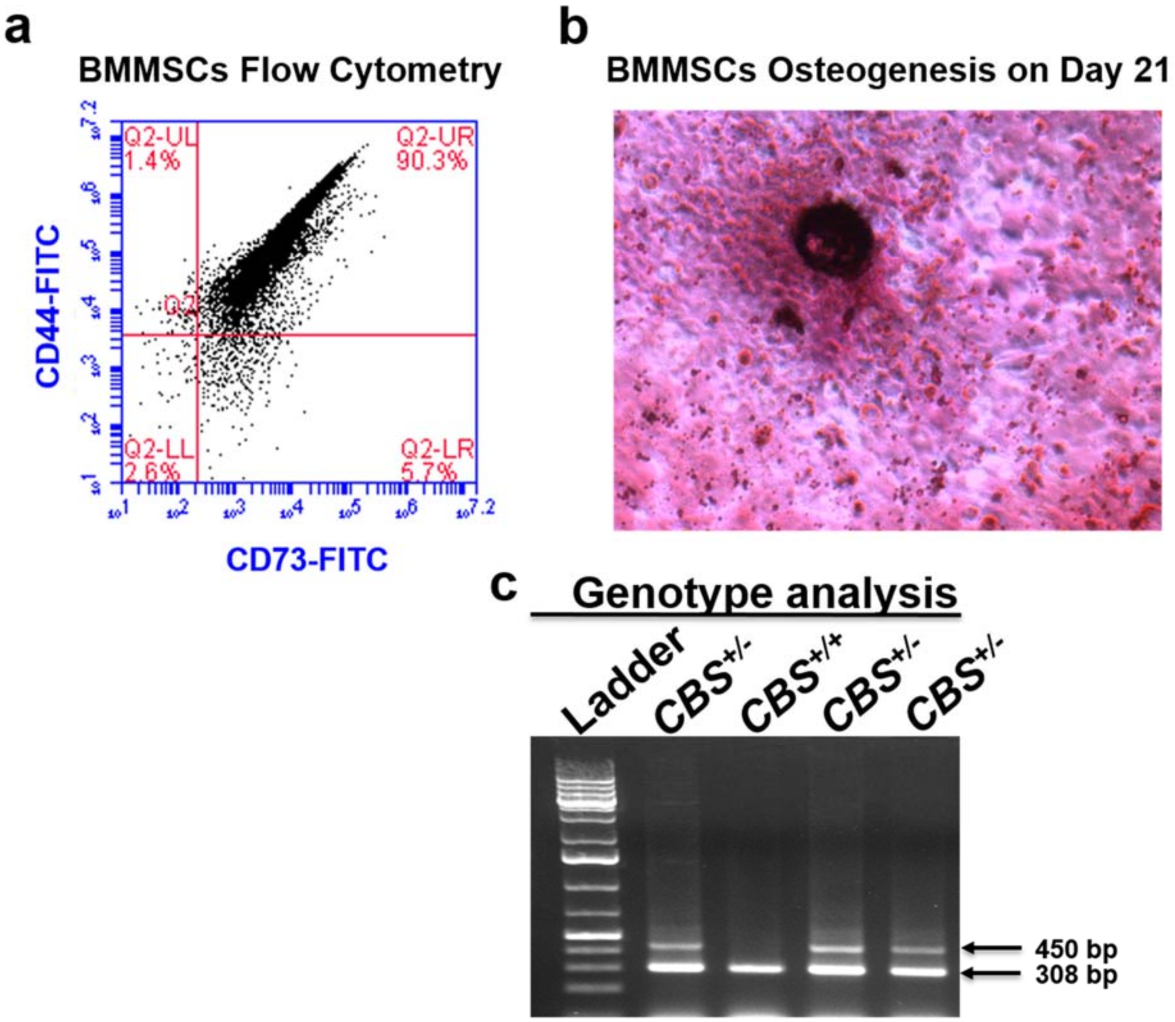

Supplementary Figure 1

<u>Supplementary Fig. S1</u>: Characterization of BMMSCs. (A) Flow cytometry analysis of BMMSCs assessing the presence of CD44 and CD73. 90.3% of BMMSCs were stained positive for both CD44 and CD73. (B) To evaluate BMMSCs differentiation and mineralization, BMMSCs were plated at a concentration of 3.2x10^2/well and were grown for 21 days in osteogenic medium. Mineralization capacity was evaluated by Alizarin red staining. (C) Representative genotype image of CBS mice done with specific sets of primers. Heterozygote CBS$^{+/-}$mice produced two bands of 450 and 308 bp; CBS$^{+/+}$ mice produced a single band of 308 bp. Experiments were repeated three times.

Full-length blots for Figure 1

Fig.1b: CBS

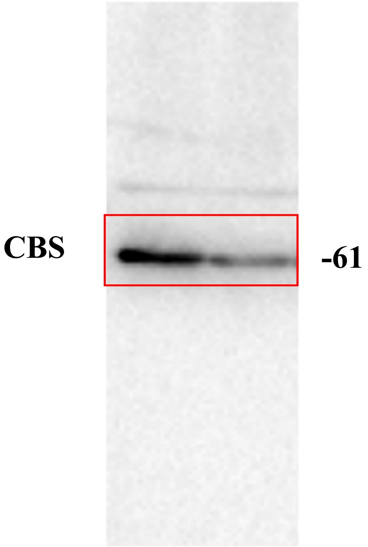

Fig.1b: GAPDH

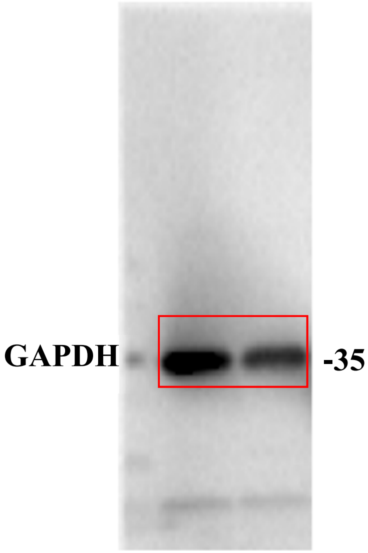

Supplementary Fig. S2: Uncropped gels/blots for main Fig.1b. Boxed regions are used in the main figures. For some data, transferred membranes were horizontally cut for applying multiple antibodies. Lanes for unrelated samples are deleted. Lanes from left to right in red box: WT, $CBS^{+/-}$.

Full-length blots for Figure 2

Fig.2c: HO-1

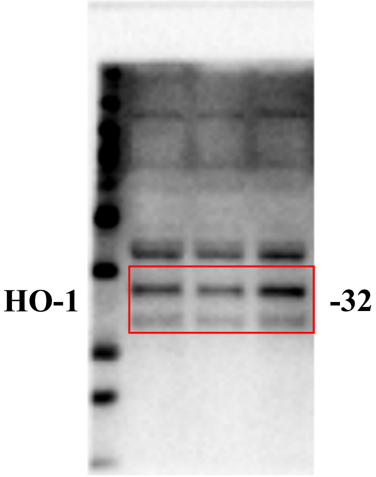

Fig.2c: 4-HNE

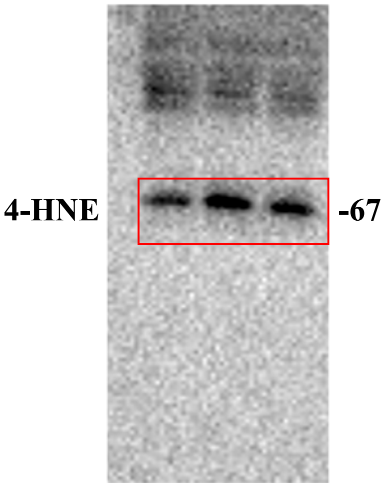

Fig.2c: GAPDH

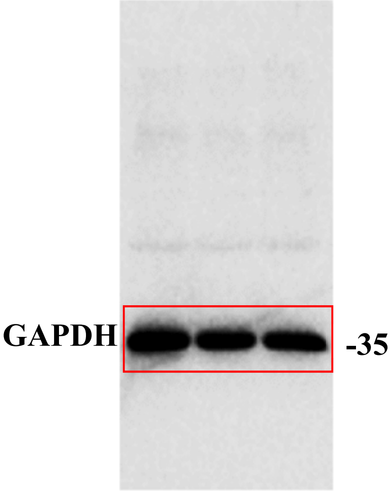

<u>Supplementary Fig. S3</u>: Uncropped gels/blots for main Fig.2c. Boxed regions are used in the main figures. For some data, transferred membranes were horizontally cut for applying multiple antibodies. Lanes for unrelated samples are deleted. Lanes from left to right in red box: WT, *CBS*$^{+/-}$, *CBS*$^{+/-}$+NaHS.

<u>Full-length blots for Figure 3</u>

Fig.3a: H3K27ac **Fig.3a: HDAC3** **Fig.3a: H3** **Fig.3a: GAPDH**

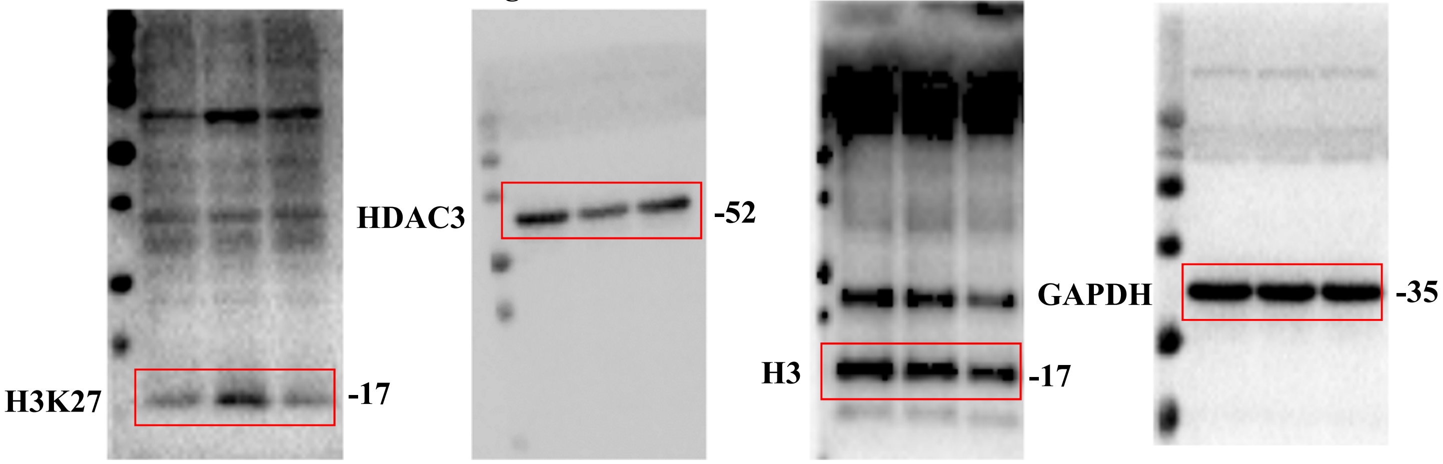

<u>Supplementary Fig. S4</u>: Uncropped gels/blots for main Fig.3a. Boxed regions are used in the main figures. For some data, transferred membranes were horizontally cut for applying multiple antibodies. Lanes for unrelated samples are deleted. Lanes from left to right in red box: WT, *CBS*$^{+/-}$, *CBS*$^{+/-}$+NaHS.

Fig.3g: ChIP IL-6 **Fig.3g: ChIP TNF-α** **Fig.3g: Input DNA**

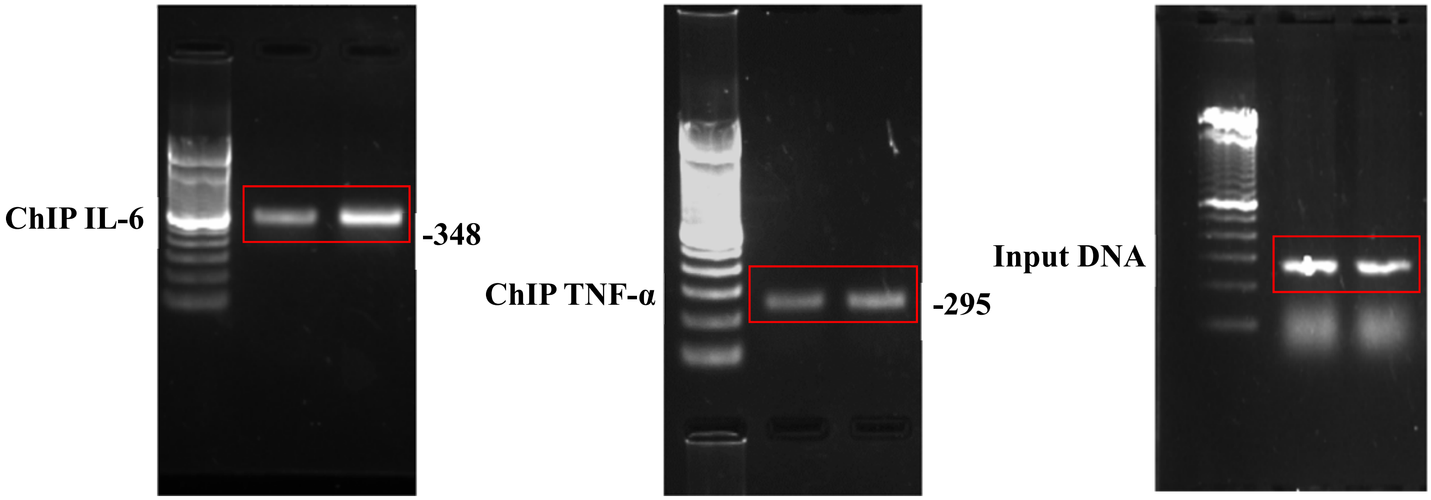

Supplementary Fig. S5: Uncropped gels/blots for main Fig.3g. Boxed regions are used in the main figures. For some data, transferred membranes were horizontally cut for applying multiple antibodies. Lanes for unrelated samples are deleted. Lanes from left to right in red box: WT, *CBS*$^{+/-}$, *CBS*$^{+/-}$+NaHS.

Fig.3j: ChIP RunX2

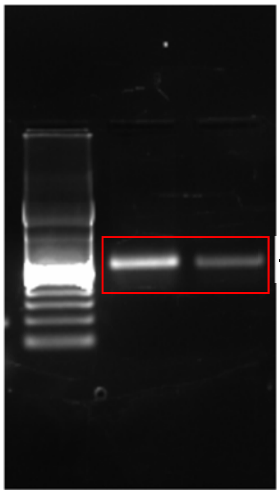

Fig.3j: ChIP OCN

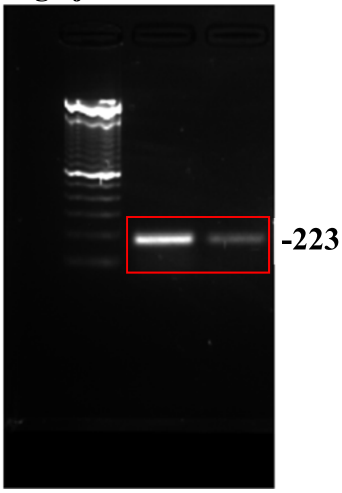

Fig.3j: Input DNA

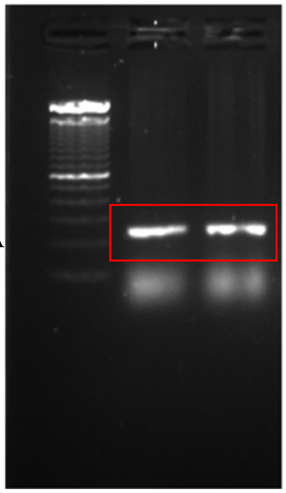

Supplementary Fig. S6: Uncropped gels/blots for main Fig.3j. Boxed regions are used in the main figures. For some data, transferred membranes were horizontally cut for applying multiple antibodies. Lanes for unrelated samples are deleted. Lanes from left to right in red box: WT, *CBS*$^{+/-}$, *CBS*$^{+/-}$+NaHS.

Full-length blots for Figure 4

Fig.4a: NF-KbP65

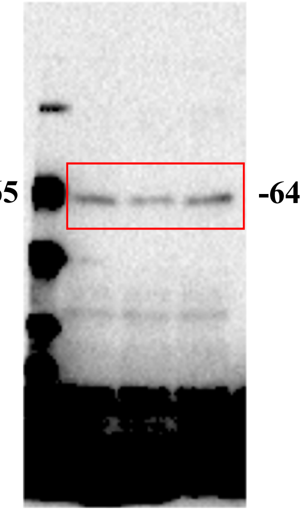

Fig.4a: NF-KbP65 (Ac-K310)

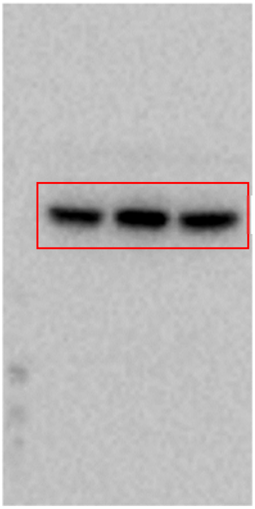

Fig.4a: GAPDH

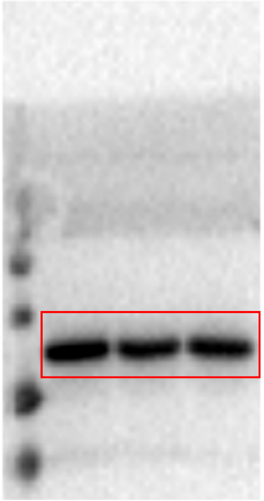

<u>Supplementary Fig. S7</u>: Uncropped gels/blots for main Fig.4a. Boxed regions are used in the main figures. For some data, transferred membranes were horizontally cut for applying multiple antibodies. Lanes for unrelated samples are deleted. Lanes from left to right in red box: WT, *CBS*$^{+/-}$, *CBS*$^{+/-}$+NaHS.

Full-length blots for Figure 6

Fig.6h: OCN

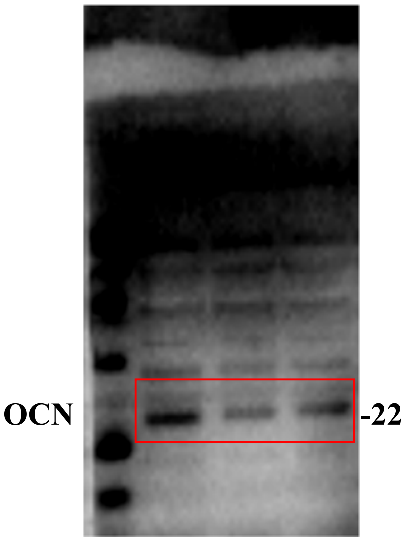

Fig.6h: RUNX2

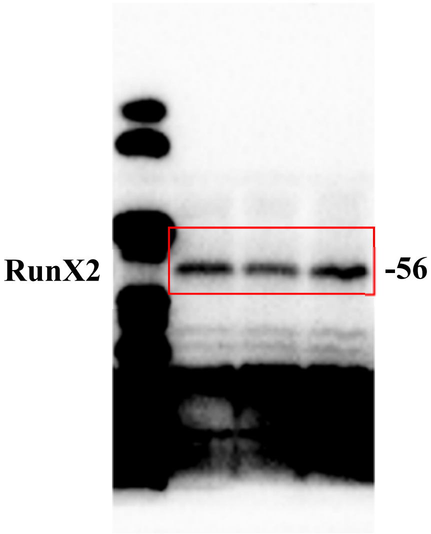

Fig.6h: GAPDH

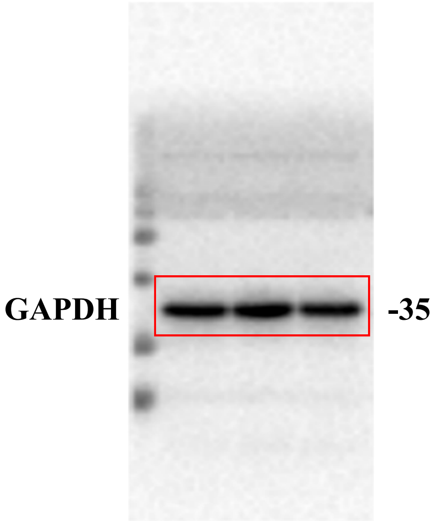

<u>Supplementary Fig. S8</u>: Uncropped gels/blots for main Fig.6h. Boxed regions are used in the main figures. For some data, transferred membranes were horizontally cut for applying multiple antibodies. Lanes for unrelated samples are deleted. Lanes from left to right in red box: WT, *CBS*$^{+/-}$, *CBS*$^{+/-}$+NaHS.

Full-length blots for Figure 7

Fig.7s: SHY-RUNX2

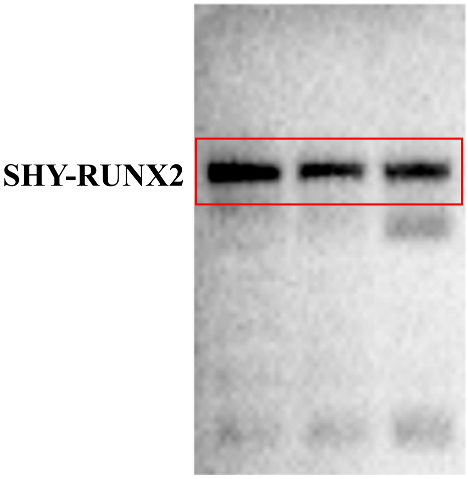

Fig.7s: Input DNA

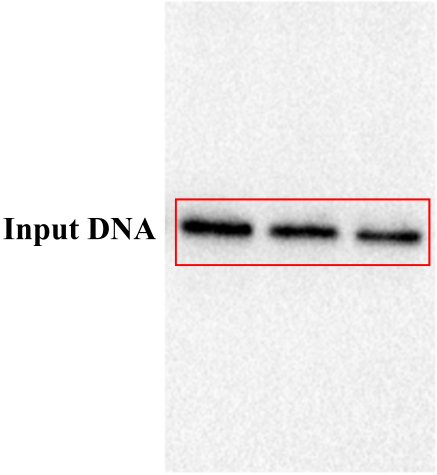

Supplementary Fig. S9: Uncropped gels/blots for main Fig.7s. Boxed regions are used in the main figures. For some data, transferred membranes were horizontally cut for applying multiple antibodies. Lanes for unrelated samples are deleted. Lanes from left to right in red box: WT, *CBS*$^{+/-}$, *CBS*$^{+/-}$+NaHS.

Fig.7u: SHY-RUNX2

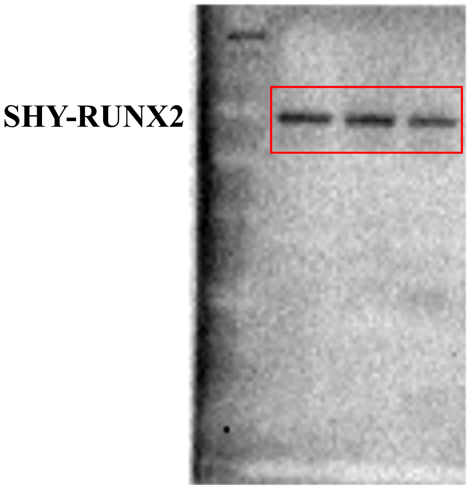

Fig.7u: Input DNA

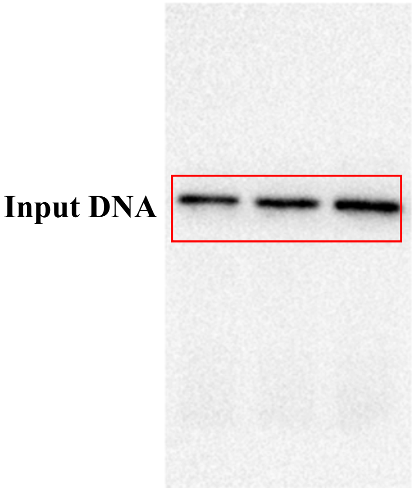

Supplementary Fig. S10: Uncropped gels/blots for main Fig.7u. Boxed regions are used in the main figures. For some data, transferred membranes were horizontally cut for applying multiple antibodies. Lanes for unrelated samples are deleted. Lanes from left to right in red box: NaHS, NaHS + DTT, DTT.
